# Small G protein signalling modulator 2 (SGSM2) is involved in oestrogen receptor-positive breast cancer metastasis through enhancement of migratory cell adhesion via interaction with E-cadherin

**DOI:** 10.1080/19336918.2019.1568139

**Published:** 2019-02-11

**Authors:** Juo-Han Lin, Wen-Jui Lee, Han-Chung Wu, Chih-Hsiung Wu, Li-Ching Chen, Chi-Cheng Huang, Hang-Lung Chang, Tzu-Chun Cheng, Hui-Wen Chang, Chi-Tang Ho, Shih-Hsin Tu, Yuan-Soon Ho

**Affiliations:** a Ph.D. Program for Cancer Molecular Biology and Drug Discovery, College of Medical Science and Technology, Taipei Medical University and Academia Sinica, Taipei, Taiwan; b Ph.D. Program for Neural Regenerative Medicine, College of Medical Science and Technology, Taipei Medical University and National Health Research Institutes, Taipei, Taiwan; c Institute of Cellular and Organismic Biology, Academia Sinica, Taipei, Taiwan; d Department of Surgery, School of Medicine, College of Medicine, Taipei Medical University, Taipei, Taiwan; e Department of General Surgery, En Chu Kong Hospital, New Taipei City, Taiwan; f Breast Medical Center, Taipei Medical University Hospital, Taipei, Taiwan; g Taipei Cancer Center, Taipei Medical University, Taipei, Taiwan; h TMU Research Center of Cancer Translational Medicine, Taipei Medical University, Taipei, Taiwan; i School of Medicine, College of Medicine, Fu-Jen Catholic University, New Taipei City, Taiwan; j Department of Surgery, Fu-Jen Catholic University Hospital, New Taipei City, Taiwan; k School of Medical Laboratory Science and Biotechnology, College of Medical Science and Technology, Taipei Medical University, Taipei, Taiwan; l Department of Laboratory Medicine, Taipei Medical University Hospital, Taipei, Taiwan; m Department of Food Science, Rutgers University, New Brunswick, NJ, USA; n Graduate Institute of Medical Sciences, College of Medicine, Taipei Medical University, Taipei, Taiwan

**Keywords:** SGSM, breast cancer, cell adhesion, E-cadherin, oestrogen receptor, EMT

## Abstract

The function of small G protein signalling modulators (SGSM1/2/3) in cancer remains unknown. Our findings demonstrated that SGSM2 is a plasma membrane protein that strongly interacted with E-cadherin/β-catenin. SGSM2 downregulation enhanced the phosphorylation of focal adhesion kinase (FAK; Y576/577), decreased the expression of epithelial markers such as E-cadherin, β-catenin, and Paxillin, and increased the expression of Snail and Twist-1, which reduced cell adhesion and promoted cancer cell migration. Oestrogen and fibronectin treatment was found to promote the colocalization of SGSM2 at the leading edge with phospho-FAK (Y397). The BioGRID database showed that SGSM2 potentially interacts with cytoskeleton remodelling and cell-cell junction proteins. These evidences suggest that SGSM2 plays a role in modulating cell adhesion and cytoskeleton dynamics during cancer migration.

## Introduction

A distinct metastatic pattern is one characteristic of breast cancer (BC); metastasis often occurs in the lymph nodes, bone, lung, and liver, leading to a high death rate. Tumour cell metastasis and invasion are complicated processes involving multiple steps; recently, the most commonly described step of metastasis initiation is the epithelial-mesenchymal transition (EMT) [1]. EMT is a complex process that occurs in wound healing, organ fibrosis, or the initiation of cancer metastasis. During this process, cells first lose cell-cell adhesion through disruption of E-cadherin-mediated junctions, and then increased cell motility via interplay between the actin cytoskeleton and cell adhesion sites leads to the generation of membrane protrusions and traction forces [2–5]. Migration inducers include fibroblast growth factor (FGF), transforming growth factor β (TGF-β), epidermal growth factor (EGF), insulin-like growth factor-1 (IGF1), tumour necrosis factor-α (TNFα), and Wnt/β-catenin, and the regulation of EMT transcription factors, such as Snail and Twist [6–9], which drive cell movement by direct repression of E-cadherin cell adhesion molecule transcription. In addition, the upregulation of proteases, such as matrix metalloproteinases (MMPs), can also disrupt E-cadherin/catenin-mediated cell-cell adhesion [10,11], and hepatocyte growth factor/scatter factor (HGF/SF), a multifunctional cytokine that is secreted by mesenchymal cells in the tumour microenvironment, can downregulate E-cadherin and induce pro-migratory small G proteins (small GTPases), such as Ras, Rho/Rac, and Ras-associated protein (Rap) [5,12,13]. The activation of small GTPases increases cell motility and migration not only through the promotion of membrane trafficking but also through the control of cytoskeletal reorganization and cell adhesion [14,15].

A novel group of small G protein signalling modulators (SGSM1/2/3) were first reported in 2007 [16]. TBC and RUN motifs are two major conserved domains of the SGSM family and appear to be related to the modulation of the small GTPases RAP and RAB, which mediate signal transduction and vesicular transportation pathways [16–18]; additionally, a RAP-interacting domain (RAPID) motif among all the SGSMs was identified as the true interaction site between SGSM and the RAP family (RAP1A/1B/2A/2B) [16]. Many studies have indicated that RAP proteins play an important role in the formation of cadherin-based cell-cell junctions, especially RAP1, which regulates adhesion and migration in many cancers via shuttling between its inactive GDP- and active GTP-bound form [19–21]. Currently, the role of SGSM proteins in cancer is still unknown, but SGSM proteins have been identified as GTPase-activating proteins (GAPs), which can modulate G protein signalling by interacting with RAP and RAB family proteins [22,23]. Therefore, this study aims to determine whether SGSM proteins participate in BC carcinogenesis and to identify the underlying mechanisms.

According to our first screening, *SGSM1* mRNA was not expressed in BC or breast epithelial cells, and compared with *SGSM2* expression, *SGSM3* expression was not different between normal breast tissue and tumour tissues based on RT-PCR. We consequentially narrowed our focus to SGSM2 and investigated its function in BC. Real-time PCR data revealed that *SGSM2* mRNA was more highly expressed in ER-positive malignant tissues than in ER-negative tissues from 200 BC patients, and its protein expression was also associated with ER-positive BC cells. Interestingly, we found that trypsin could cleave SGSM2 protein on the plasma membrane, which was confirmed by a cytosol and membrane extraction assay. This novel finding indicated that SGSM2 is a plasma membrane protein. Consistently, knockdown of *SGSM2* by small interfering RNA (siRNA) induced the phosphorylation of focal adhesion kinase (FAK; Y576/577), a decrease in the expression of the epithelial markers E-cadherin, β-catenin, and Paxillin, and an increase in the expression of upstream epithelial markers Snail and Twist-1, which led to a reduction in cell adhesion and the promotion of cancer cell migration. In addition, SGSM2 was found to exhibit a strong interaction with E-cadherin/β-catenin cell junction complexes, even in the presence of EGTA (4 mM), which inhibits the formation of this complex, and in the presence of EGF (100 nM), which induces E-cadherin endocytosis. SGSM2 was also found to participate in oestrogen- and fibronectin-induced cell migration, and colocalization with phospho-FAK (Tyr397) was clearly observed at the leading edge at the beginning of cell migration. The prediction from the BioGRID database showed that SGSM2 potentially interacts with cytoskeleton remodelling and cell-cell junction proteins, including formin-binding protein 1-like (FNBP1L), Wiskott-Aldrich syndrome-like (WASL), cell division cycle 42 (CDC42), and cadherin 1 (CDH1). These novel findings demonstrate that SGSM2 may be involved in the modulation of cell adhesion and cytoskeleton dynamics through an E-cadherin-mediated EMT process during the initial stage of cancer migration.

## Results

### SGSM2 mRNA expression was associated with luminal a breast cancer rather than HER2-enriched or basal-like breast cancer

**Figure 1.**
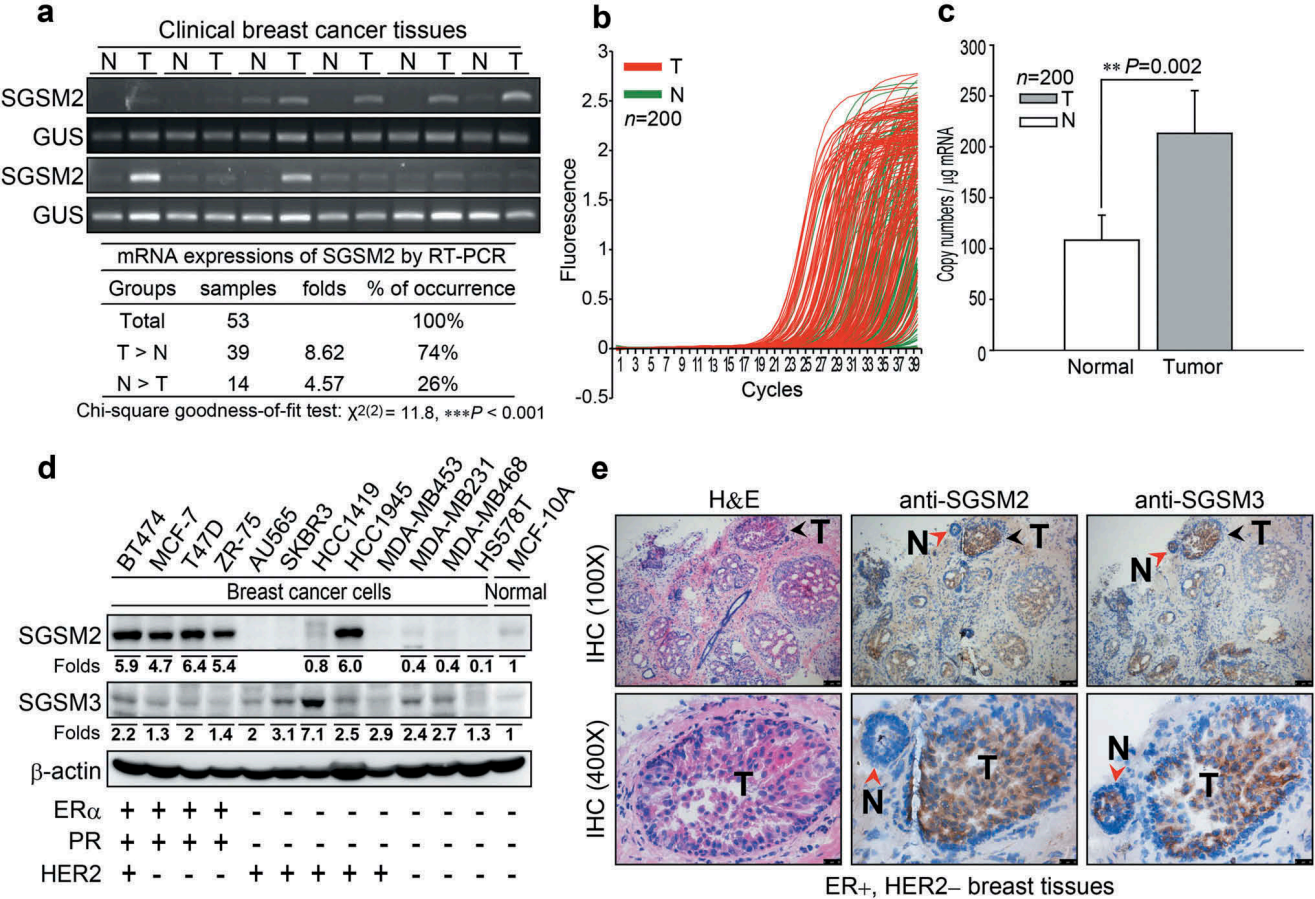
*SGSM2* expression was detected in human breast tissues and human breast cancer cell lines. (a) *SGSM2* mRNA expressions in normal and malignant tissues was determined in 53 BC patients via RT-PCR, and the gel band intensities were quantified with PhotoCaptMw software (version 11.0.). We used normalized *SGSM2* expression to classify patients into three groups: T > N, N = T, and N > T (N = normal tissue, and T = tumour tissue); and further, (b) quantitative real-time PCR was used to evaluate *SGSM2* mRNA expression profiles in paired human breast tumour (red lines) and normal (green lines) tissues (n = 200). (c) The means of *SGSM2* mRNA copy number (per μg of mRNA) were calculated from real-time PCR data. The data were analysed with a paired sample *t*-test with two-sided P-values. (d) *In vitro* SGSM2 and SGSM3 protein expression was detected in twelve breast cancer cell lines and one breast epithelial cell line; the mean density of SGSM2 and SGSM3 protein level in each cell line was normalized to β-actin, and the values were compared to the MCF-10A cell line. The hormone receptor status is displayed at the bottom. (e) The distribution of SGSM2 and SGSM3 proteins were determined in ER+/ HER2 – breast cancer tissue sections; Haematoxylin (violet, for nuclei) and eosin (pink) (H&E) staining is shown in the left panels; anti-SGSM2 and anti-SGSM3 antibody bound to antigen is shown in the middle and right panels (N = normal region, T = tumour region). Top panels: 100x, scale bar: 100 μm; bottom panels: 400x, scale bar: 25 μm.

To determine whether *SGSM2* expression correlated with BC, we randomly detected the *SGSM2* mRNA level in 53 BC sample tissues via RT-PCR, as shown in Figure 1(a). Among 53 BC patients, 74% had *SGSM2* mRNA expression in tumours that was higher than that in normal tissue (T > N, n = 39), but in 26% of patients, *SGSM2* mRNA expression in tumour tissue was less than that in normal tissue (N > T, n = 14). The mean of the fold difference in the T > N group (8.62-fold) was higher than that in the N > T group (4.57-fold) (Figure 1(a), Chi-square goodness-of-fit test, ***P < 0.001). We further evaluated *SGSM2* mRNA in 200 paired normal and malignant breast tissues using real-time PCR (Figure 1(b,c). *SGSM2* expression was observed more often in early cycles in tumour tissues (red lines) than in normal tissues (green lines) (Figure 1(b)), and the average *SGSM2* copy number in paired tumour tissues was 2-fold higher than that in paired normal tissues (Figure 1(c), bar 2 *vs*. bar 1 = 213.2 *vs*. 108.3; **P = 0.002). Statistical analysis of the correlation between *SGSM2* and the clinical status of the tumour tissues is shown in Table 1. The copy number was transformed into log2 (copy number +1) values. *SGSM2* had significantly higher expression in ER+, PR+, HER2 – breast tumours than in ER–, PR–, HER2+ tumours (Tukey HSD test, *P = 0.046; Table 1), and an elevated *SGSM2* mRNA level was found in well-differentiated tumours (Grade 1) but not in poorly differentiated tumours (Grade 3); however, the results were non-significant (Table 1). To confirm these observations, the *SGSM2* mRNA level obtained using RNAseq data of the TCGA Breast Cancer (BRCA) cohort via UCSC Xena browser (http://xena.ucsc.edu) was calculated (Supplementary Table 1). The *SGSM2* mRNA level correlated with ER+, PR+, and HER2 – BC (***P < 0.001; Supplementary Table 1), and increased *SGSM2* mRNA expression was predominately detected in tissue samples from patients with luminal A type BC compared with HER2-enriched and basal-like BC patients (Scheffe test, ***P < 0.001). Box plots showing *SGSM2* mRNA levels associated with ER status and PAM50 subtype are provided in Figure S1(a-d).

**Table 1.** Clinical *SGSM2* mRNA expression status was detected with real-time PCR in tumour samples.

Factors	*SGSM2* Copy number (Mean ± S.E.M)	Statistics P-value	Case Number	Factors	*SGSM2* Copy number (Mean ± S.E.M)	Statistics P-value	Case Number
**ER status**	Student’s t-test	P *= *0.320	Total = 152	**Radiotherapy**	Student’s t-test	P *= *0.070	Total = 96
Negative positive	4.74 ± 0.41		45	non-treatment	4.95 ± 0.34		75
	5.27 ± 0.29		107	post-treatment	3.65 ± 0.56		21
**PR status**	Welch’s t-test	P *= *0.129	Total = 151	**Chemotherapy**	Welch’s t-test	P = 0.979	Total = 108
Negative positive	4.74 ± 0.29		68	non-treatment	5.11 ± 0.51		35
	5.45 ± 0.36		83	post-treatment	5.09 ± 0.38		73
**HER2 status**	Student’s t-test	P *= *0.121	Total = 134	**Hormonotherapy**	Student’s t-test	P *= *0.674	Total = 111
Negative positive	5.64 ± 0.32		94	non-treatment	4.93 ± 0.48		37
	4.76 ± 0.44		40	post-treatment	5.20 ± 0.37		74
**Menopause**	Student’s t-test	P *= *0.968	Total = 61	**Target therapy**	Student’s t-test	P *= *0.629	Total = 91
No	6.48 ± 0.81		16	non-treatment	5.46 ± 0.37		77
Yes	6.52 ± 0.48		45	post-treatment	5.00 ± 0.87		14
**Receptor subtype**	ANOVA	P *= *0.070	Total = 134	**Grade**	ANOVA	No Homogeneity	Total = 144
ER+PR+HER2-	5.78 ± 0.38	Tukey HSD	72	Grade 1	4.97 ± 0.43		37
ER+PR+HER2+	5.65 ± 0.66		21	Grade 2	5.50 ± 0.38		76
ER-PR-HER2+	3.77 ± 0.20	* 0.046	19	Grade 3	4.32 ± 0.42		31
Triple negative	5.17 ± 0.53		22				
**Stage**	ANOVA	P *= *0.623	Total = 158	**N status**	ANOVA	P *= *0.822	Total = 158
stage 0	3.80 ± 0.73		3	N0	5.21 ± 0.36		72
stage I	5.58 ± 0.51		37	N1	4.91 ± 0.35		54
stage II	4.91 ± 0.30		82	N2	5.86 ± 1.18		9
stage III	5.51 ± 0.63		27	N3	5.22 ± 0.76		19
stage IV	4.46 ± 1.81		5				
**T status**	ANOVA	P *= *0.690	Total = 155	**M status**	Student’s t-test	P *= *0.896	Total = 157
T1	5.25 ± 0.43		52	M0	5.01 ± 0.24		144
T2	5.10 ± 0.31		89	M1	4.88 ± 0.84		9
T3	4.63 ± 0.83		9				
T4	2.00 ± 0.00		1				

The copy number is displayed as the log2 (copy number +1) value. Independent t-tests (Welch’s t-test for heterogeneous variances) were used to compare differences between two groups, and one-way ANOVA (Tukey HSD test) was used to compare differences among three or more groups. Quantitative data are presented as the mean ± S.E.M; the mean is the average log2 (*SGSM2* copy number +1) value. All P-values are two-tailed, and * indicates statistical significance with P ≤ 0.05.

### SGSM2 protein expression in ER-positive breast cancer

Next, we examined SGSM2 and SGSM3 protein expression in 12 BC cell lines (BT474, MCF-7, T-47D, ZR-75, AU565, SKBR3, HCC1419, HCC1945, MDA-MB453, MDA-MB231, MDA-MB468, and HS578T) and one breast epithelial cell line (MCF-10A) (Figure 1(d)). Compared with SGSM3, SGSM2 was not generally expressed in all cells; it appeared to be primarily expressed in ER-positive BC cells (BT474, MCF-7, T-47D, and ZR-75) and HCC1945 (Figure 1(d)). Positive SGSM2 staining in tumours compared with normal cells was confirmed by IHC in ER+/HER2 – BC tissue sections (Figure 1(e), middle panels). However, SGSM3 was homogenously expressed in both tumour and normal tissues (Figure 1(e), right panels). These preliminary data revealed that SGSM2 expression was stronger in BC with the ER-positive phenotype.

### Trypsin-EDTA disrupted SGSM2 protein expression on the plasma membrane

**Figure 2.**
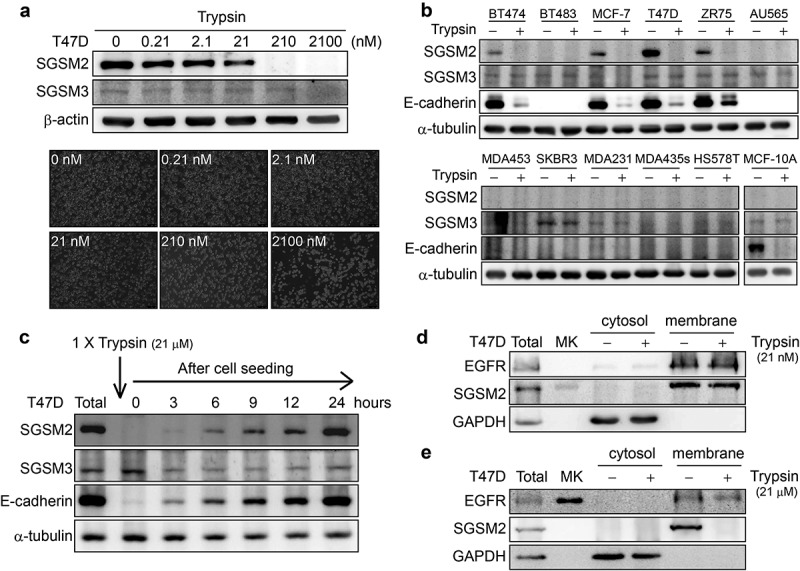
Trypsin-EDTA-digested SGSM2 protein on the cell membrane in the T47D breast cancer cell line. (a) T47D cells were treated with different concentrations (0, 0.21, 2.1, 21, 210, or 2100 nM) of trypsin-EDTA for 3 minutes; bottom graphs show different cell morphologies after trypsin-EDTA treatment (5x, scale bar: 250 μm). (b) SGSM2, E-cadherin, and SGSM3 protein expression changes were observed in 11 adherent breast cell lines and a human melanoma cell line (MDA-MB-435s) after treatment with or without trypsin-EDTA (21 μM). (c) T47D cells were treated with trypsin-EDTA (1X; 21 μM) for subculture, and then, SGSM2, E-cadherin, and SGSM3 protein levels were observed at different time points (0, 3, 6, 9, 12, and 24 hours) after cell seeding. (d) Cytosol and membrane extracts were examined to confirm SGSM2 protein localization after 21 nM trypsin-EDTA treatment, and (e) higher (21 μM) trypsin-EDTA concentrations were further examined. ‘Total’, representing total protein, which was the positive control, was extracted after cell seeding for 48 hours. GAPDH and EGFR were used as the positive cytosol and membrane controls, respectively. α-Tubulin served as the internal control for western blotting.

The typical trypsin-EDTA concentration used to detach cells adhered to culture dishes is ~21 μM. Because SGSM2 protein was incidentally found to be digested by trypsin-EDTA, to verify this observation, adherent T47D cells were treated with different trypsin concentrations (0, 2.63, 5.25, 10.5, and 21 μM) for 3 minutes (Figure S2(a)). These concentrations were sufficiently high to destroy SGSM2 protein; therefore, we diluted the trypsin-EDTA concentration to 0, 0.21, 2.1, 21, 210, or 2100 nM; the results shown in Figure 2(a). Interestingly, SGSM2 protein disappeared when the cells were treated with trypsin-EDTA at concentrations higher than 21 nM; however, SGSM3 protein expression did not exhibit concentration-dependent differences (Figure 2(a)). Similar results were obtained using another protease (chymotrypsin). Treatment with chymotrypsin at several concentrations (1, 5, 10, 50, and 100 μg/ml) also completely degraded SGSM2 protein within 5 minutes (Figure S2(c,d)). Next, we treated a panel of adherent breast cancer cell lines and a human melanoma cell line (MDA-MB-435 s) with or without 21 μM trypsin-EDTA and analysed SGSM2 protein by western blot (Figure 2(b)). SGSM2 protein nearly vanished after trypsin treatment, and E-cadherin, a cell membrane marker, was also affected by trypsin; only SGSM3 remained present (Figure 2(b)). Next, we demonstrated that SGSM2 protein was significantly regenerated 6 hours after cell seeding when using 1X (21 μM) trypsin-EDTA, and E-cadherin was renewed at an earlier time (Figure 2(c)). Notably, the formation of 55-kDa SGSM2 protein fragments exhibited a time-dependent decrease after trypsin-EDTA treatment of T47D cells (Figure S2(e)). Trypsin can breakdown the proteins responsible for surface adherence during cell passaging; thus, cytosol and membrane extraction experiments were performed to clarify whether SGSM2 protein was located at the plasma membrane. Surprisingly, we found that SGSM2 was present in the plasma membrane along with EGFR, while both were absent from the cytosolic extract (Figure 2(d,e). Furthermore, compared with a low-dose (21 nM) trypsin treatment, a high dose (21 μM) of trypsin disturbed this phenomenon (Figure 2(d *vs*. e). The above evidence reliably confirms that SGSM2 is located on the plasma membrane, and our study is the first to report such an observation.

### Treatment of T47D cells with trypsin affected cell adhesion ability

**Figure 3.**
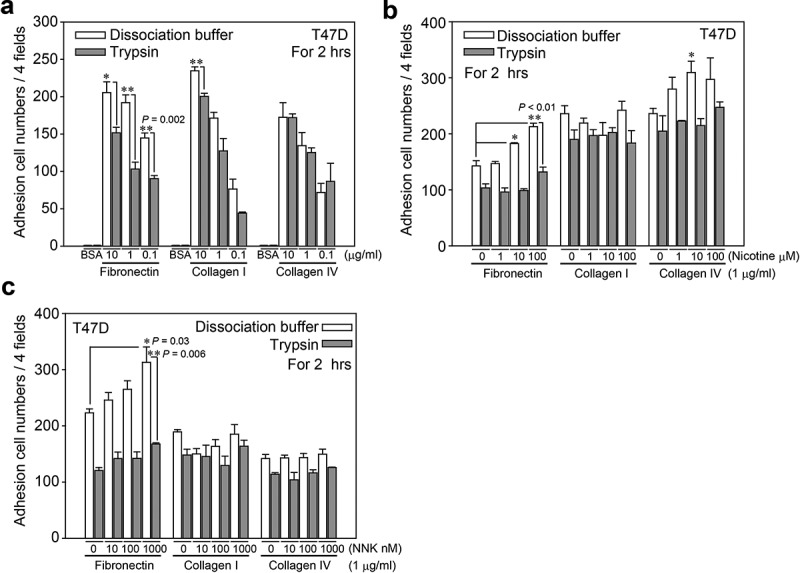
The cell adhesion ability of T47D cells on different ECM proteins was determined. (a) Using different concentrations (0.1, 1, and 10 μg/ml) of three types (fibronectin, type I, and type IV collagens) of ECM proteins, cell adhesion ability between cell dissociation buffer- and trypsin-EDTA (1X; 21 μM)-detached T47D cells was assessed. The BSA group was the negative control. Finally, we chose a 1 μg/ml dose of ECM proteins for the following experiments. (b) (c) Different concentrations (0, 1, 10, and 100 μM) of nicotine and NNK (0, 10, 100, and 1000 nM) were used to determine the influence the adhesion ability of dissociation buffer- and trypsin-detached T47D cells cultured on three types of ECM proteins (1 μg/ml). P-values were determined with Student’s *t*-test; *P < 0.05 and **P < 0.01. All experiments were repeated more than 3 times (n > 3).

Because SGSM2 is a plasma membrane protein, its function needs to be clarified. We first selected the optimal concentration of extracellular matrix (ECM) proteins, such as fibronectin, type I collagen, and type IV collagen, for cell adhesion assays. Then, an enzyme-free cell dissociation buffer rather than trypsin was used to detach T47D cells to ensure that the SGSM2 protein structure was not affected. Figure 3(a) shows that the most significant difference between treatment with dissociation buffer and treatment with trypsin occurred in the fibronectin-coated group, especially at concentrations of 0.1 and 1 μg/ml fibronectin (**P = 0.002, and **P < 0.01); therefore, 1 μg/ml fibronectin was chosen to coat the dish for the following experiments. Nicotine and NNK have been documented as a co-carcinogen and carcinogen, respectively, that promote cancer formation and increase the risk of cancer invasion [24,25]. Enhancement of focal adhesion ability is one characteristic that facilitates cancer cell migration. Next, we wished to clarify whether trypsin treatment would affect nicotine- or NNK- induced cell adhesion. We harvested cells with cell dissociation buffer and found that the average number of adherent cells was dose-dependently (0, 1, 10, 100 μM) increased in the fibronectin (1 μg/ml) group after treatment with nicotine (Figure 3(b), bar 1 *vs*. 7 and bar 7 *vs*. 8; **P < 0.01, bar 1 *vs*. 5; *P = 0.013). However, this result was not observed after treating the cells with trypsin. The same phenomenon was observed after treatment with different doses (0, 10, 100, 1000 nM) of NNK (Figure 3(c), bar 1 *vs*. 7; *P = 0.03, bar 7 *vs*. 8; **P = 0.006), and the result was more significant for exposure to NNK than for exposure to nicotine.

### Calebin A decreased SGSM2 protein levels and inhibited cancer cell adhesion ability

**Figure 4.**
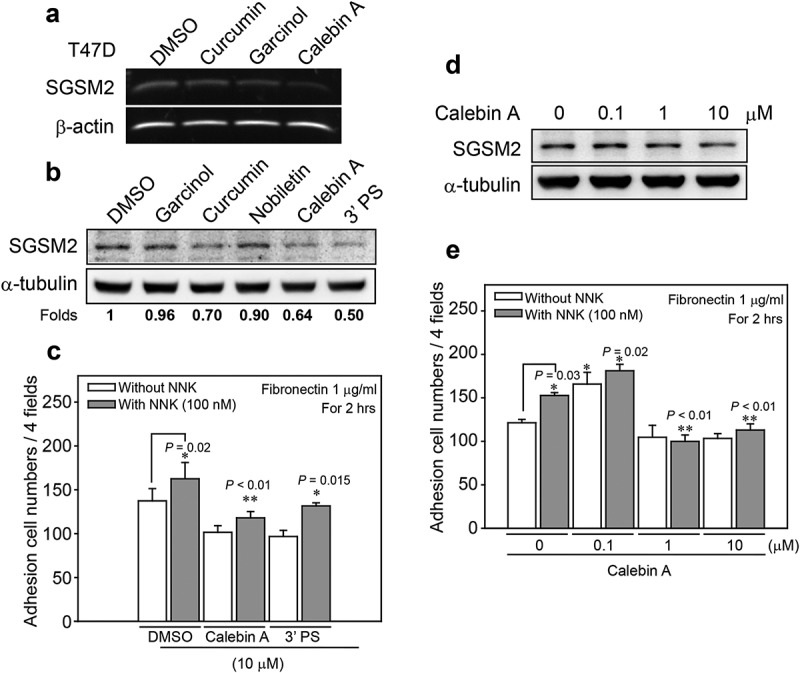
Natural compounds inhibited SGSM2 protein expression and decreased NNK-induced cell-matrix adhesion. (a) Determination of *SGSM2* mRNA levels after treatment with 10 μM of several natural compounds (curcumin, garcinol, and calebin A) for 24 hours (b) Determination of the SGSM2 protein levels after treatment with 10 μM of various natural compounds (garcinol, curcumin, nobiletin, calebin A, and 3’ PS) for 24 hours; DMSO was the solvent control. The mean density of SGSM2 protein level in each cell line was normalized to α-tubulin, and the values were compared with DMSO. (c) Differences in T47D cell adhesion abilities were investigated by treatment with two (calebin A and 3’ PS; 10 μM) natural compounds combined with or without NNK (100 nM). (d) SGSM2 proteins were dose-dependently decreased by treatment with calebin A (0, 0.1, 1, and 10 μM) for 24 hours. (e) NNK (100 nM)-induced cell adhesion abilities were significantly inhibited by cotreatment with 1 and 10 μM calebin A. For adhesion experiments, 1 μg/ml fibronectin was coated onto the dish. α-Tubulin was the internal control. P-values were determined with Student’s *t*-test; *P < 0.05, and **P < 0.01. All experiments were repeated more than 3 times (n > 3).

According to the above-described data, trypsinization of adherent cells may disrupt SGSM2 protein on the plasma membrane (Figure 2) and decrease cell adhesion ability (Figure 3). However, because trypsin exposure affects not only SGSM2 protein but also other cell membrane proteins, we screened several natural compounds for the specific inhibition of SGSM2 protein. *SGSM2* mRNA levels were decreased by treatment with 10 μM calebin A for 24 hours, and the results are shown in Figure 4(a). Figure 4(b) shows that calebin A and 3’ PS (10 μM) more strongly repressed SGSM2 protein. The cell adhesion ability in the calebin A (10 μM) combined with NNK (100 nM) group was significantly suppressed compared with the DMSO group (Figure 4(c), bar 2 vs. 4; **P < 0.01). Additionally, 3’ PS (10 μM) treatment also inhibited the NNK-induced increase in cell adhesion ability (Figure 4(c), bar 2 vs. 6; *P = 0.015). T47D cells were further treated with different concentrations (0, 0.1, 1, or 10 μM) of calebin A for 24 hours. Calebin A at 1 µM was able to inhibit SGSM2 protein levels; however, treatment with 10 μM calebin A produced more significant results (Figure 4(d)). Furthermore, cotreatment with NNK and 1 μM or 10 μM calebin A significantly suppressed NNK-induced cell adhesion (Figure 4(e), bar 2 *vs*. 6; **P = 0.003, bar 2 *vs*. 8; **P = 0.007).

### SGSM2 *silencing decreased breast cancer cell adhesion and promoted cell migration*

**Figure 5.**
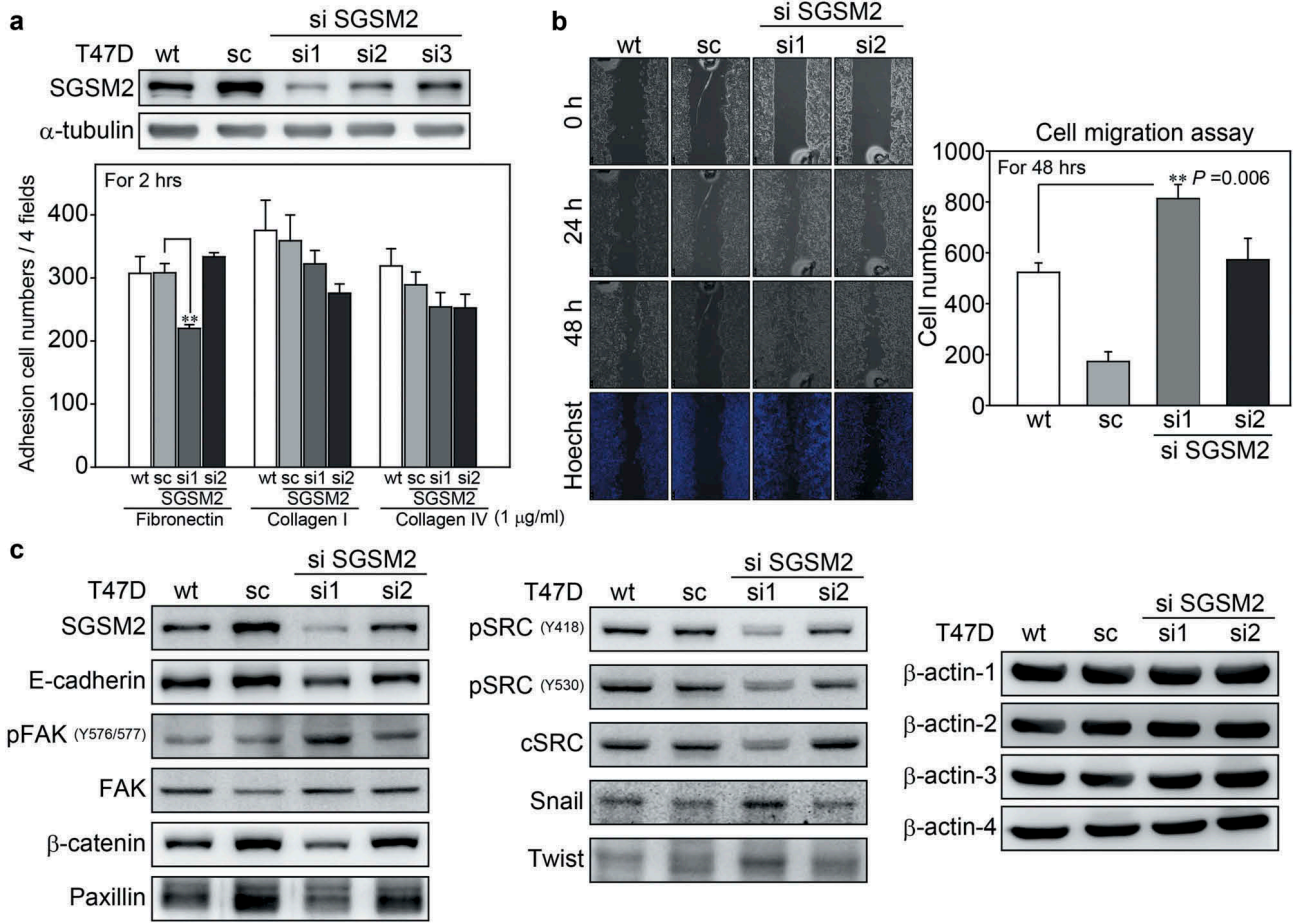
*SGSM2* silencing affected T47D cell adhesion and cell migration. (a) The efficiency of SGSM2 knockdown was determined by western blotting (upper panel); sc (scramble), si1, si2, and si3 *SGSM2* stable cell lines were collected by G418 selection. The lower panel shows the adhesion abilities of different stable cell lines plated on three types (fibronectin, type I collagen, and type IV collagen; 1 μg/ml) of ECM proteins. Wt indicates wild-type T47D cells. (b) Cell migration abilities of four types (wt, sc, si1, si2) of T47D cells were examined with wound-healing assays; left panels show the cell migration states at 0, 24, and 48 hours. Hoechst was used to stain the nuclei. The average number of migrated cells is presented in the right panel. (c) The protein levels of E-cadherin, β-catenin, Paxillin, FAK, SRC, Snail, and Twist were detected by western blotting in wt, sc, si1, and si2 T47D cells. β-Actin-1, 2, 3, 4 served as the internal controls. P-values were determined with Student’s *t*-test; **P < 0.01. All experiments were repeated more than 3 times (n > 3).

Although calebin A decreased SGSM2 protein levels and inhibited NNK-induced cell adhesion (Figure 4), these findings do not demonstrate that SGSM2 is involved in regulating cancer cell adhesion. We next established stable *SGSM2* expression, knockdown and scramble T47D cell lines. Two pSUPER si*SGSM2* (si1 and si2) and one pSUPER scramble colonies were selected for the following experiments (Figure 5(a), upper panel). The cell shape upon *SGSM2* silencing was observed by bright field microscopy, as shown in Figure S3. Compared with wild-type and scramble T47D cells, which exhibited a round and aggregate shape (Figure S3(a,b)), cells of the *SGSM2* si1 displayed a flattened and more spindle morphology (Figure S3(c)). In addition, the cell adhesion ability of the si2 *SGSM2* cells was not significantly different from that of wild-type or scramble cells. However, si1 *SGSM2*, with almost complete inhibition of SGSM2 expression, exhibited significantly decreased cell adhesion in the fibronectin group (Figure 5(a), sc *vs*. si1; **P < 0.01, n > 3). A wound-healing assay showed that *SGSM2* knockdown may increase BC cell migration (Figure 5(b)). The left panels show that si1 *SGSM2* cells migrated quickly at 24 hours, and at 48 hours, the cells appeared to close the wound gap. The cell numbers are shown in the right panels (Figure 5(b), wild-type (wt) *vs*. si1; **P = 0.006, n > 3). EMT is a biological process in which a non-motile epithelial cell converts to a mesenchymal phenotype with invasive capacity [26] through molecular changes that decrease cell-cell recognition and adhesion and increase the potential for cell motility [1]; therefore, changes in EMT-associated markers can be used to identify this phenomenon. Certain epithelial markers, such as E-cadherin, β-catenin, and Paxillin, were decreased in si1 *SGSM2* T47D cells. In contrast, the phosphorylation of FAK (Y576/577) was significantly increased. Snail and Twist-1 expression was also increased in si1 *SGSM2* T47D cells (Figure 5(c)). These results revealed that *SGSM2*-silenced BC cells exhibit a more migratory phenotype, decreased cell adhesion and improved migration abilities, but why SRC protein was dramatically decreased in si1 *SGSM2* T47D cells remains unknown.

### SGSM2 interacted with E-cadherin and β-catenin cell-cell adhesion molecules

**Figure 6.**
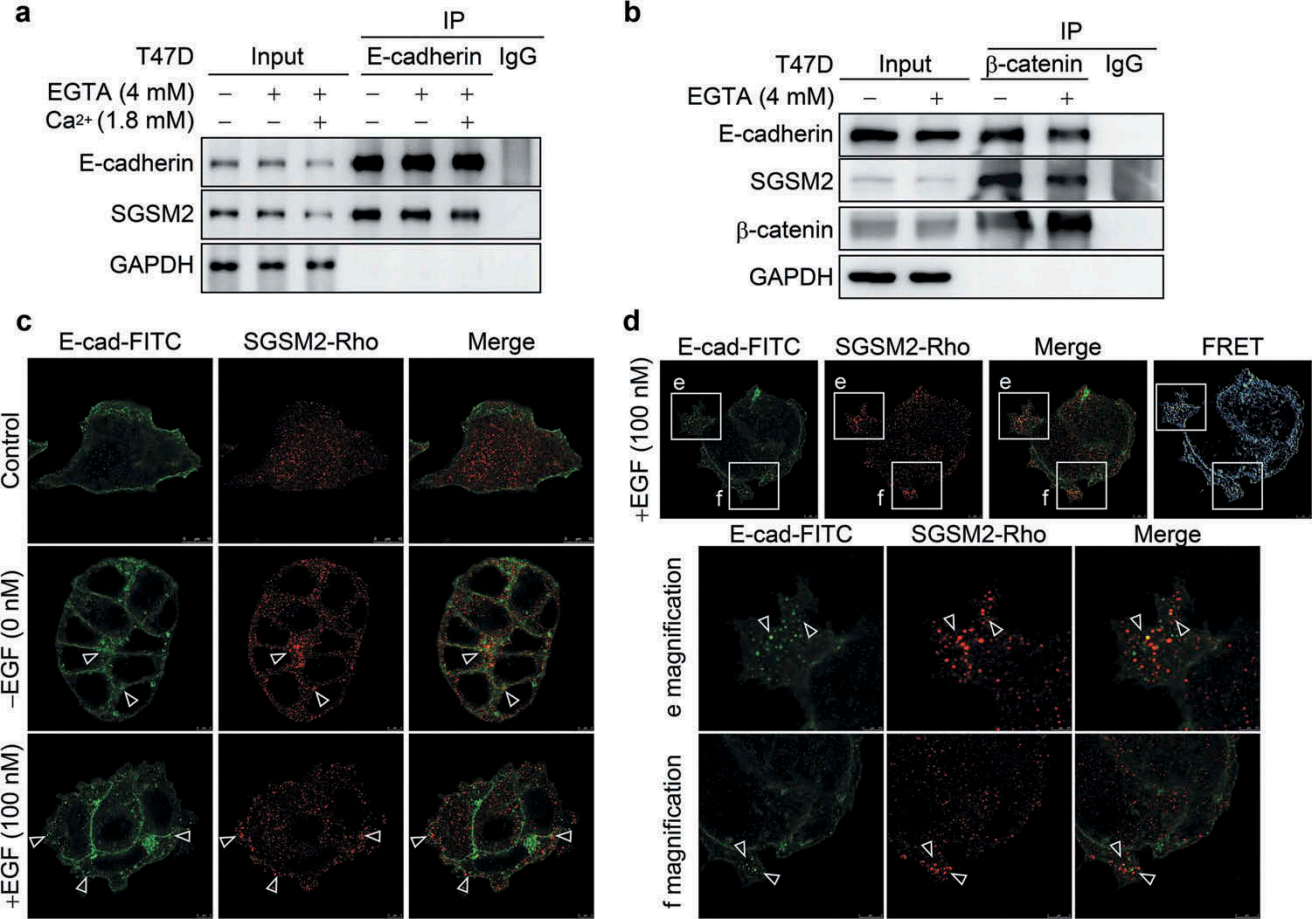
Interaction between SGSM2 and E-cadherin was verified by co-immunoprecipitation and immunofluorescence staining assays. (a) Co-IP of E-cadherin or (b) β-catenin was assessed via western blotting. T47D cells were treated with or without EGTA (4 mM); calcium ions (Ca^2+^) (1.8 mM) were further added to reform adherent junctions after treatment with EGTA for 30 minutes. Protein complexes were pulled down by anti-E-cadherin or anti-β-catenin antibody and subsequently analysed by western blotting. Irrelevant IgG was used as a negative control. (c) The colocalization of E-cadherin-FITC (green) and SGSM2-rhodamine (red) was detected by IF staining. EGF (100 nM) was used to induce E-cadherin endocytosis. Scale bar of control: 10 μm; scale bars of EGF – and EGF+: 5 μm. White arrows indicate colocalization of E-cadherin and SGSM2. (d) The colocalization of E-cadherin-FITC and SGSM2-rhodamine was determined by EGF (100 nM) treatment. FRET activity strongly indicated an interaction between SGSM2 and E-cadherin (upper panels; scale bar: 5 μm). Enlarged images of the boxed areas in e and f are shown in the bottom panels. Scale bar of e magnification: 2.5 μm; scale bars of magnifications: 5 μm.

During the process of EMT, cells become spindle-shaped and exhibit increased intercellular separation, increased motility, and loss of cell-cell adhesion [27]. An E-cadherin/β-catenin/αE-catenin complex, also called CCC, is present in the plasma membrane and is involved in cell-cell adhesion [28]. Based on our above results, we considered that the function of SGSM2 might correlate with cell-cell junctions. EGTA, a Ca^2+^ -specific chelator, can disrupt cadherin-mediated cell-cell adherens junctions, and subsequent restoration of Ca^2+^ ions may re-establish cadherin-mediated cell-cell contacts [29]. Thus, we used these two molecular approaches to perform the immunoprecipitation (IP) experiments. When we pulled down E-cadherin, SGSM2 was also precipitated, which indicated that E-cadherin and SGSM2 have a strong interaction, and EGTA (4 mM) and calcium (Ca^2+^; 1.8 mM) ion addition subsequent to EGTA did not affect this interaction (Figure 6(a)). Because β-catenin can conjugate with E-cadherin, we further performed IP with β-catenin and found that SGSM2 could also be brought down with β-catenin, even in the presence of EGTA (4 mM) treatment (Figure 6(b)). However, EGTA still had a small effect on the interaction of E-cadherin, β-catenin, and SGSM2 proteins. EGF has been reported to induce focal adhesion disassembly and enhance tumour cell motility through EGFR-activated E-cadherin endocytosis [30,31], and SGSM2 has been reported to interact with RAB family proteins, who function in recycling of vacuoles between the early endosome and plasma membrane [16]. Therefore, we investigated whether SGSM2 is involved in E-cadherin protein endocytosis. In the control T47D cells shown in Figure 6(c) (upper panels), SGSM2 (red) and E-cadherin (green) proteins on the cell adhesion surface strongly interacted, and at cell-cell adhesion junction fields, very strong colocalization between SGSM2 and E-cadherin was found (Figure 6(c), middle panels; arrowheads). This phenomenon was still observed during 100 nM EGF-induced endocytosis of E-cadherin, as shown in Figure 6(c) (bottom panels; arrowheads) and Figure 6(d) (E and F magnifications; arrowheads). These results were convincing because of the strong Fluorescence resonance energy transfer (FRET) activity observed (Figure 6(d)). To clarify whether SGSM2 is located in endocytic vesicles after EGF treatment, we also performed IF staining using early endosome antigen 1 (EEA1), which is an early endosome marker and SGSM2 antibody. The results indicated positive colocalization of SGSM2 with EEA1 with or without EGF treatment, as shown in Figure S4.

### SGSM2 colocalized with phospho-FAK (Y397) at oestrogen-stimulated focal adhesions and ruffling surfaces

**Figure 7.**
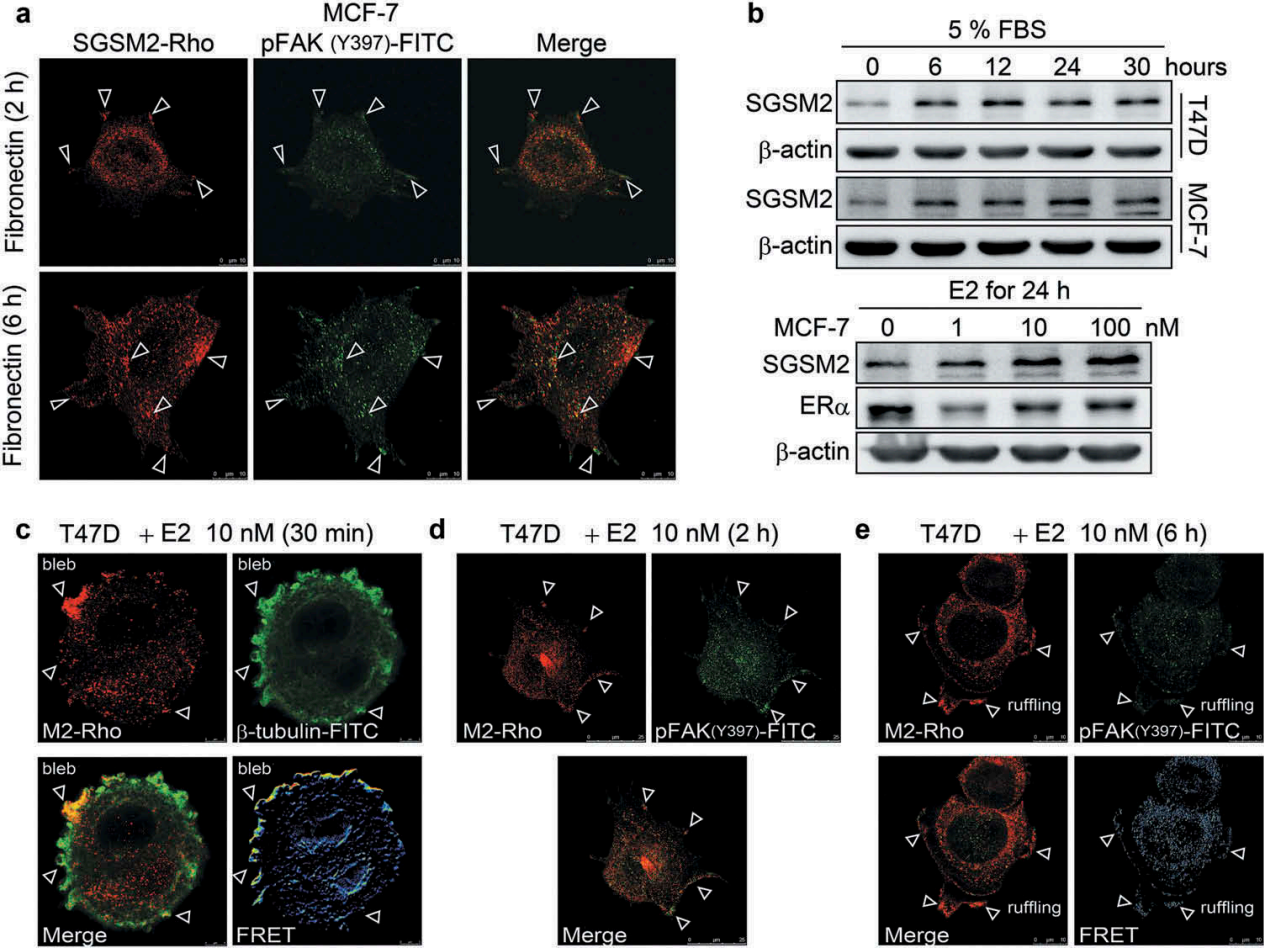
SGSM2 was located with p-FAK (Y397) at the leading edge of oestrogen-induced migrating cells. (a) SGSM2 and p-FAK(Y397) colocalized at focal adhesions in fibronectin-stimulated MCF-7 cells. The upper images were acquired at the 2-hour mark after cell seeding on fibronectin-coated slides; the bottom image was captured at the 6-hour mark after cell seeding. SGSM2 is shown in red (rhodamine), and p-FAK (Y397) is shown in green (FITC). White arrows indicate colocalization of SGSM2 and p-FAK (Y397) at focal adhesion sites. Scale bar, 10 μm. (b) SGSM2 protein expression was induced in T47D and MCF-7 cells at different times (0, 6, 12, 24, and 30 hours) by 5% FBS (serum) exposure (upper panel). SGSM2 was dose-dependently (0, 1, 10, 100 nM) increased in MCF-7 cells by treatment with oestradiol (E2) (bottom panel). β-Actin was the internal control. (c) Cell bleb formation occurred at the 30-minute mark after E2 (10 nM) exposure. SGSM2 is shown in red (rhodamine), and β-tubulin is shown in green (FITC). A strong interaction was indicated by FRET activity. White arrows indicate colocalization of SGSM2 and β-tubulin. Scale bar, 5 μm. (d) Focal adhesion formation occurred at the 2-hour mark after E2 (10 nM) exposure; white arrows indicate colocalization of SGSM2 and p-FAK (Y397) at focal adhesion sites. Scale bar, 25 μm (e) Cell ruffling formation occurred at the 6-hour mark after E2 (10 nM) exposure; white arrows indicate colocalization of SGSM2 and p-FAK (Y397) at ruffling sites. Scale bar, 10 μm. (d) (e) SGSM2 is shown in red (rhodamine), and p-FAK (Y397) is shown in green (FITC).

To study nascent adhesion formation, cell dissociation buffer was used to harvest MCF-7 cells, which were then placed onto fibronectin-coated glass slides in the absence of serum to synchronize adhesion site formation. After cell seeding for 2 hours, SGSM2 was found to colocalize with phospho-FAK (Y397) at initial adhesion sites (Figure 7(a); upper panels, arrowheads). Particularly at the 6-hour mark of cell seeding, this colocalization became more visible (Figure 7(a); bottom panels, arrowheads). A 3D image showed that FAK obviously colocalized with SGSM2 on fibronectin-coated glass slides (Figure S5(a)). Because oestrogen was reported to promote endothelial cell and BC cell motility through ERα-activated FAK phosphorylation and the subsequent formation of focal adhesion complexes [32,33], we suggested that SGSM2 protein was expressed relative to ER expression in BC cells (Figure 1(d)). Here, 5% foetal bovine serum (FBS)-induced SGSM2 protein production was determined at the sixth hour after treatment in both T47D and MCF-7 cells (Figure 7(b), upper panels), and we observed that SGSM2 protein levels were dose-dependently (0, 1, 10, 100 nM) increased in MCF-7 cells by 17β-oestradiol (E2) treatment (Figure 7(b), bottom panels). We next studied SGSM2 protein localization during oestrogen-induced focal adhesion formation. Changes in cellular motility resulted in membrane blebbing, which was observed at the beginning of treatment with E2 (10 nM) for 30 minutes. FRET activity revealed that SGSM2 (red) strongly colocalized with β-tubulin (green) in the plasma membrane blebs of T47D cells (Figure 7(c), arrowheads), and disruption of the membrane-actin cortex was observed (Figure S5(b)). At the second hour of treatment with E2 (10 nM), phospho-FAK^39,7^ (green) and SGSM2 (red) were distributed at the leading edge (Figure 7(d), arrowheads). Moreover, FRET activity also showed that phospho-FAK^39,7^ (green) and SGSM2 (red) were colocalized at the E2-induced ruffles formed at the plasma membrane during the sixth hour (Figure 7(e), arrowheads). This evidence indicates that both fibronectin (1 μg/ml) and E2 (10 nM) treatment could induce SGSM2 and phospho-FAK^39,7^ colocalization during the dynamic remodelling of the cytoskeleton and cell membrane and that SGSM2 may be involved in cell motility regulation.

### Higher SGSM2 mRNA levels were found in ER-positive metastatic patients than in those with an ER-negative status

Our study verified that inhibition of SGSM2 could reduce cell adhesion and increase cancer cell migration; however, we also found that SGSM2 was involved in modulating E2-induced cell motility. These two results appear contradictory; thus, we analysed the possible effect of *SGSM2* gene expression in BC patients in the TCGA Breast Cancer (BRCA) cohort. Higher *SGSM2* expression was found in metastatic tumour tissues than in primary site tumours (Figure S6(a,b)). We further analysed pathologic Meta status compared with ER status in primary site tumours and observed that *SGSM2* expression correlated only with ER-positive BC patients but not with ER-negative patients (Figure S6(c,d); M1: ER+ *vs*. ER–, **P = 0.009 and D; M1: ER+ *vs*. ER–, *P = 0.015). These data definitively confirmed that SGSM2 is involved in oestrogen-induced cancer cell migration and is regulated by the ER pathway.

## Discussion

In 1999, scientists created the ‘full-length long Japan’ (FLJ) collection of sequenced human cDNAs and first reported the complete sequencing and characterization of 21,243 human cDNA clones in 2004 [34]. Hence, three novel proteins (*SGSM1/2/3*) were first identified for their function in modulating the small G protein (RAP and RAB)-mediated signalling pathway in 2007 [16]. In this study, we first determined the *SGSM1/2/3* mRNA expression in BC and normal breast cell lines; however, only *SGSM2* and *SGSM3* were found to be expressed in these cells, and no *SGSM1* expression was observed. This finding was consistent with a previous report that indicated that *SGSM1* is expressed mainly in the brain, heart, and testis [16]. We further investigated *SGSM2* and *SGSM3* mRNA levels in tumours and paired normal tissues from 53 BC patients via RT-PCR. Compared with *SGSM3* expression, *SGSM2* expression in tumours was significantly higher, and its percentage of occurrence in the T > N group was 74%. Based on quantitative real-time PCR, *SGSM2* gene expression in paired tumour tissues was 2-fold higher than that in paired normal tissues from 200 BC patients, and this expression significantly correlated with luminal A type BC rather than the HER2-positive or TNBC types. These data provided evidence that was confirmed not only with our clinical statistics but also using the TCGA Breast Cancer (BRCA) cohort. Moreover, SGSM2 protein was highly expressed in ER-positive BC cells (BT474, MCF-7, T-47D, and ZR-75) and in ER-positive breast tumour tissues.

Currently, there are still few studies on SGSM2. One report indicated that SGSM2, also known as RUTBC1, is a Rab9A effector that can promote the GTP hydrolysis of Rab32 and Rab33B proteins [22] and demonstrated that it regulates Rab9A-mediated melanogenic enzyme trafficking in melanocytes through a GAP for Rab32/38 [18,22,35,36]. These findings indicate that SGSM2 may be involved in transport from late endosomes to the trans-Golgi network; however, it has not been clarified whether SGSM2 is located on the endomembrane system. Incidentally, we noticed that during the cell culture process, 21 μM trypsin-EDTA could cleave SGSM2 protein, and we confirmed that SGSM2 protein was cleaved by treatment with trypsin-EDTA at concentrations of 21 nM or higher. This phenomenon was found not only in all SGSM2-expressing BC cells but also in E-cadherin-expressing cells. We further observed that an approximately 55-kDa fragment of SGSM2 formed after 1X trypsin-EDTA treatment and verified that SGSM2 is a plasma membrane protein via a cytosol and membrane extraction assay. According to previous reports and our findings, we proposed that if SGSM2 is a membrane protein, it might also be located in the endomembrane system and involved in membrane trafficking pathways in the cell. Although trypsinization can induce proteome alterations due to the up- or downregulation of protein expression [37], our results showed that high doses of trypsin (210 and 2100 nM) did not affect *SGSM2* mRNA levels (Figure S2(b)). However, SGSM2 protein disappeared from the membrane extracts after exposure to high-dose (≥21 μM) trypsin but not low-dose (<21 nM) trypsin. These data verified that trypsin directly digests SGSM2 on the cell surface, but SGSM2 is not affected by trypsin-induced changes in protein expression.

Consistently, the cell adhesion ability of trypsinized T47D cells was significantly decreased compared with non-trypsinized cells on plates coated with different fibronectin concentrations (0.1, 1, and 10 μg/ml), but these results did not occur on type I- and type-II collagen. In addition, trypsinization also inhibited nicotine- and NNK-induced cell adhesion ability on fibronectin. These results revealed that trypsinization reduced cell adhesion by abolishing anchor molecules on fibronectin but not type I- and type-II collagen substrates. Natural compounds, such as garcinol, curcumin, and nobiletin, have been reported to exhibit anti-metastatic activity through the regulation of EMT-related protein expression [38–42]. We next found that curcuminoids, such as calebin A and 3’ PS, which inhibit SGSM2 protein expression, suppressed the NNK-induced increase in cell adhesion on a 1 μg/ml fibronectin-coated dish. We also found that SGSM2 protein was slightly increased by 0.1 μM calebin A treatment but was decreased after 1 and 10 μM calebin A treatment. These results are consistent with the cell adhesion data in which 0.1 μM calebin A combined with NNK increased T47D cell adhesion ability; however, high-dose (1 and 10 μM) calebin A significantly inhibited cell adhesion ability regardless of whether it was combined with NNK. Although there are few studies indicating an inhibitory effect of calebin A on cancer metastasis, we here verified its ability to suppress cancer cell adhesion, and this activity might be associated with SGSM2 protein expression. Furthermore, we observed the T47D si1 cell line, with greater SGSM2 silencing, exhibited a greater capacity to inhibit adhesion (**P < 0.01) than the T47D si2 clone cell line, which exhibited slight SGSM2 inhibition. Additionally, *SGSM2* knockdown promoted T47D cell migration, which was associated with the upregulation of p-FAK (Y576/577) and epithelial-associated markers (Snail and Twist) and the downregulation of E-cadherin, β-catenin, and Paxillin. By contrast, E-cadherin, β-catenin, and Paxillin were slightly increased in scramble cell lines in which SGSM2 was upregulated, and the cell migration ability was significantly inhibited.

SGSM2 functions as a modulator of RAP proteins, and we know that the RAP family is involved in various cellular phenomena, such as integrin-mediated cell adhesion, cadherin-mediated cell junction formation, and actin dynamics [16]. Our data revealed that SGSM2 knockdown might trigger cell migration by altering the expression of cell-cell junction proteins and inhibiting cell adhesion. Therefore, we hypothesized that SGSM2 is associated with E-cadherin endocytosis. EGTA disrupts E-cadherin-mediated intercellular junctions and induces E-cadherin endocytosis, and this phenomenon is restored by treatment with Ca^2+^[29]. In our co-IP experiments, E-cadherin and SGSM2 strongly interacted with each other regardless of pretreatment EGTA-induced cell endocytosis formation or the reformation of adherens junctions by further incubation with Ca^2+^. The same result was observed when a β-catenin antibody was used in a pull-down assay. This evidence demonstrated that SGSM2 directly interacts with E-cadherin/β-catenin.

EGF induces cell migration and invasion via caveolin-dependent E-cadherin endocytosis and downregulation of caveolin-1 and E-cadherin expression [31]. Under normal conditions, SGSM2 colocalized with E-cadherin at cell junctions. After EGF treatment for 30 minutes, SGSM2 also colocalized with E-cadherin during EGF-induced endocytosis. Strong energy transfer was observed in the FRET activity assay, demonstrating that SGSM2 might interact with E-cadherin at the cell surface junction and be involved in E-cadherin endocytosis. The ECM protein fibronectin has been shown to enhance the metastatic potential of BC cells [43], and it can stimulate FAK phosphorylation through integrin-mediated signalling pathways during tumour cell migration and invasion [44]. After seeding MCF-7 cells on fibronectin-coated slides and allowing them to adhere for two hours, slight SGSM2 localization with p-FAK (Y397) was observed at the fibronectin-induced focal adhesion position, and this phenomenon became more apparent at the sixth hour. The conspicuous colocalization between FAK and SGSM2 was also observed in the 3D image.

Next, we confirmed that 5% FBS and E2 exposure can stably induce SGSM2 protein expression. Oestrogen has been reported to promote BC cell motility and invasion via FAK phosphorylation, and FAK recruits the small GTPase cdc42, which induces N-WASP phosphorylation [33]. Ligand-induced ERα interacts with the G protein Gα_13_, leading to activation of the small GTPase RhoA and the downstream effector Rho-associated kinase, resulting in myosin phosphorylation and driving actin remodelling [45]. Bleb formation is driven by cell contractions, increases the intracellular pressure and has been determined to occur at the leading edge of moving cells. Three components of the cytoskeleton (microfilaments, microtubules, and intermediate filaments) are required for regulating blebbing, and myosin II contraction in the actin cortex, which is regulated by the small GTPase RhoA, leads to membrane detachment and bleb inflation [46,47]. In the dynamic changes in motility induced by E2, abundant SGSM2 was observed to accumulate with β-tubulin at the plasma membrane blebs at the 30-minute mark; moreover, colocalization of SGSM2 and p-FAK (Y397) was found not only in focal adhesions but also at the leading edge of ruffling and lamellipodia. Herein, we demonstrated that SGSM2 is involved in EGF-induced E-cadherin endocytosis and is associated with E2-induced cancer cell migration, and SGSM2 might be involved in regulation of cytoskeleton dynamics. Interestingly, the BioGRID database, which can be used to predict the physical interaction between SGSM2 and other proteins, was verified by our results. Four of the sixteen physical interactions identified, namely, interactions with CELSR3, EGFR, FNBP1L, and MMP1, were associated with cell migration and invasion (Figure S7(a)), and it is also noteworthy that FNBP1L was found to interact with WASL, CDC42, and CDH1, which are involved in cytoskeleton remodelling and cell-cell junctions (Figure S7(b-d)). This prediction is consistent with our presumption, but additional data are still needed to further support these findings in future work.

Finally, website databases were used to confirm our findings. Because SGSM2 and E-cadherin proteins were shown to be highly associated in ER-positive BC cells, we considered that they present similar clinical pathological features in BC. Unexpectedly, *SGSM2* mRNA levels showed a higher distribution in invasive lobular carcinoma (ILC) than in infiltrating ductal carcinoma (IDC) (Figure S8(a), ***P < 0.001). These data were not consistent with *CDH1* expression data, which was more highly expressed in IDC than in ILC (Figure S8(b), ***P < 0.001), and even in *CDH1*-positive BC patients, a significant correlation with *SGSM2* was not found (Figure S9(a,b)). However, we observed that one common characteristic of *SGSM2* and *CDH1* is that both are highly expressed in fibroadenoma (Figure S9(c), *P = 0.002 and D, *P = 0.038). Although fibroadenoma might have the potential to develop into invasive *in situ* carcinoma in women with the *BRCA1* gene mutation [48], the overall risk of fibroadenoma progression to breast carcinoma is very low. In addition, fibroadenoma is a benign breast tumour with proliferation of both epithelial and stromal components, and TCGA data showed higher *SGSM2* gene expression in normal breast tissues than in breast tumour tissues (Figure S6(a)). Therefore, we examined the relationship between *SGSM2* and *CDH1* expression in normal breast tissues in a TCGA cohort using a Pearson correlation test and partial correlation test. A significant positive correlation between *SGSM2* and *CDH1* is shown in paired normal breast tissues [Sig. (r) = ***(.725)] but not in paired tumour tissues [Sig. (r) = (.115)] or in all breast tumours [Sig. (r) = ***(−.184)] (Supplementary Table 2). Moreover, *ERBB2* had the strongest association with *SGSM*2 expression in normal breast tissues [Sig. (r) = ***(.877)], and it appears to be an important factor influencing the relationship between *SGSM2* and *CDH1* (Supplementary Table 2; partial correlation test). More interestingly, the migration-associated markers *SNAI1, TWIST1, PIK3CA*, and *VIMENTIN* displayed a significantly negative relationship with SGSM2 in normal breast tissues, but this relationship was not found in breast tumour tissues (Supplementary Table 2). Because BC is a heterogeneous disease that is caused by DNA mutations or epigenetic changes, we conjectured that these phenomena could disrupt the connection between *SGSM2* and other genes in breast tumours, especially the *ERBB2* gene.

Consistently, *SGSM2* exhibited significantly lower expression levels in BC patients with *TP53, BRCA2*, or *PIK3R1* (PIK3CA suppressed domain) mutation (Figure S10(a), ***P < 0.001; B, ***P < 0.001 and C, *P = 0.037), and higher levels of *SGSM2* in BC patients were significantly associated with a low risk of recurrence (Figure S11(a), **P *= *0.002, CI = 59.92, risk groups hazard ratio = 1.82). Moreover, the ability of the combination of *SGSM2* with *ERBB2* or *MKI67* to predict recurrence-free survival was significantly improved (Figure S11(d), ***P *= *0.00019 and E, ***P *= *3.52e-05). High *ERBB2* or *MKI67* levels along with a low *SGSM2* level in BC patients indicated a high risk of recurrence. In previous experiments, we observed that *SGSM2* had two-fold higher expression in tumours than in normal control tissues from Taiwanese BC patients, but the above statistical data strongly indicated that lower *SGSM2* levels in BC patients could increase tumour recurrence. Although there appears to be two opposing results, according to our clinical analysis, *SGSM2* expression represents a good prognostic phenotype, such as ILC and luminal A type BC. Additionally, a functional assay revealed that loss of SGSM2 could enhance cancer cell migration. Therefore, SGSM2 might be a mediator to maintain the equilibrium of cell motility and might play a tumour suppressor role during BC formation initiation.

## Conclusions

Our study reveals that SGSM2 not only interacts with E-cadherin but also participates in cell focal adhesions and cell motility. SGSM2 is a small G protein modulator that mediates the RAP-signalling pathway and RAB-associated intracellular vesicle transportation [16], and many GTPases, including RhoA, Ras, and Rap1, play an important role in managing cell polarization to drive cell shape changes and motility [29,49]. Thus, we hypothesize that SGSM2 is involved in E-cadherin endocytosis, cell adhesion, and migration, likely through cooperation with small GTPases. Although additional evidence is needed to further clarify this hypothesis, our novel findings indicate that SGSM2 is a plasma membrane protein that coordinates cell adhesion and migration.

## Material and methods

### Tissue collection

Two hundred human BC tissues were obtained from anonymous donors at Taipei Medical University Hospital and Cathay General Hospital, Taipei, Taiwan. The study methodologies conformed to the standards set by the Declaration of Helsinki, and the study methodologies were approved by the TMU-Joint Institutional Review Board (TMU-JIRB, 20170119). Each sample was divided into paired tumour and normal tissues.

### Cell culture

Human mammary gland epithelial adenocarcinoma cell lines (luminal subtype A: BT-483 (ATCC- HTB-121), MCF-7 (ATCC HTB-22), T47D (ATCC HTB-133), and ZR75-1 (ATC CRL-1500); luminal subtype B: BT-474 (ATCC HTB-20); HER2 subtype: AU-565 (ATCC CRL-2351), SKBR3 (ATCC HTB-30), HCC1419 (ATC CRL-2326), HCC1945 (ATC CRL-2338), and MDA-MB-453 (ATCC HTB-131); basal subtype: MDA-MB-231 (ATCC HTB-26), MDA-MB-468 (ATCC HTB-132), and Hs-578T (ATCC HTB-126)), a human melanoma cell line (MDA-MB-435s), and non-tumourigenic immortalized breast epithelial cell line [MCF-10A (ATCC CRL-10317)] were purchased from American Tissue Cell Culture collection (ATCC). MCF-10A cells were maintained under conditions that have been well-established by Xiao-guang Sun et al. [50]. The other cell lines were maintained in DMEM or DMEM/F12 (Thermo Fisher Scientific) supplemented with 10% FBS, Thermo Fisher Scientific), penicillin (100 units/mL), streptomycin (100 μM/mL), and 250 μg/mL amphotericin B at 37°C in a humidified 5% CO_2_ incubator.

### Quantitative real-time PCR and reverse transcription-polymerase chain reaction (RT-PCR)

Total RNA was extracted from human cell lines and frozen breast tissue specimens using an isothiocyanate-phenol-chloroform procedure according to the TRIzol® reagent manufacturer’s protocol (Invitrogen). Complementary DNA (cDNA) was synthesized from 2 µg of total RNA with 0.5 μg of oligo(dT) primers using a reverse transcription kit (ReverTra Ace®; Toyobo). PCR amplification was performed in a 25-μl volume using Blend Taq® -Plus- kit (Toyobo) according to the manufacturer’s instructions. The conditions for the PCR were as follows: 94°C for 3 minutes; followed by 30–34 cycles of amplification at 94°C for 30 seconds, 60°C for 30 seconds and 72°C for 30 seconds; and, finally, 72°C for 7 minutes. A portion of breast tissue samples from the original group (n = 50 of 200) was randomly selected for the determination of *SGSM2* expression by RT-PCR. Furthermore, quantitative real-time RT-PCR (LightCycler® 2.0) was used to perform absolute quantitation of *SGSM2* expression, and *β-glucuronidase* (*GUS)* was used to normalize the *SGSM2* gene mRNA levels. The appropriate reagents were purchased in a preformulated kit (LightCycler FastStart DNA Master SYBR Green I; Roche Diagnostics GmbH, Roche Molecular Biochemicals). The primers for *SGSM2, SGSM3*, and the internal control *GUS* and *β-actin* were as follows: *SGSM2* forward 5’-ACACCCACTTCTACTTCTGTTATC-3’ and reverse 5’-GTCCATGTTGTTGTCACGG-3’; *SGSM3* forward 5’-GCAAGAACGACATCATCACAAT-3’ and reverse 5’-CGTCACCGAGTCATCCC-3’; *GUS* forward 5’-AGTGTTCCCTGCTAGAATAGATG-3’ and reverse 5’-AAACAGCCTGTTTACTTGAG-3’; *β-actin* forward 5’- TGTACGTTGCTATCCAGGCT-3’ and reverse 5’- CTCCTTAATGTCACGCACGA-3’.

### Immunohistochemical (IHC) analysis

ER-positive and HER2-negative frozen-embedded blocks of BC tissues were sectioned at thicknesses of 5–7 μm. IHC staining was performed as described in our pervious study [51]. The primary anti-SGSM2 antibody (1:800 dilution; NBP1-93637, Novus Biologicals) and anti-SGSM3 antibody (1:200 dilution; ab99422, Abcam) were incubated with the slides for 1 hour. Then, the signals were detected and amplified using a DAKO REAL EnVision Detection System and Peroxidase/DAB+ (LSAB2 System-HRP (K0673); Dako Corp.). Finally, histologic human BC tissue specimens were stained with haematoxylin and eosin (H&E).

### Western blot assay

For protein extraction, cells were dissociated with enzyme-free cell dissociation solution (S-014-C, Merck KGaA) instead of adherent cells with trypsin-EDTA, and they were subsequently removed with a cell scraper. The cell pellets were further lysed on ice in cell lysis buffer (20 mM Tris-HCl, pH 8.0, 137 mM NaCl, 5 mM EDTA, 5 mM EGTA, 10 mM NaF, 1% Triton X-100, and 10% glycerol) supplemented with protease inhibitors (Roche, Indianapolis, IN, USA) and phosphatase inhibitors (Sigma). The proteins were separated in 10% sodium dodecyl sulphate-polyacrylamide gel electrophoresis (SDS-PAGE) gels and transferred to polyvinylidene difluoride membranes (PVDF). The following primary mouse monoclonal antibodies were incubated with the membranes at a 1:4000 dilution for 2 hours: anti-glyceraldehyde 3-phosphate dehydrogenase (GAPDH) (sc-32233, Santa Cruz Biotechnology); and anti-β-actin and anti-α-tubulin (Santa Cruz Biotechnology). The following primary antibodies were used at a 1:1000 dilution for 2 hours: rabbit polyclonal anti-SGSM2 (NBP1-93637, Novus Biologicals); anti-β-catenin (GTX101435, GeneTex), anti-SRC, anti-SNAI1 (C15D3) (#3879, Cell Signalling Technology); goat anti-SGSM3 (ab99422, Abcam); mouse monoclonal anti-Twist (ab175430, Abcam); anti-E-cadherin (610182, BD Biosciences); and anti-FAK (sc-1688, Santa Cruz Biotechnology). The membranes were then incubated with secondary HRP-conjugated anti-mouse or anti-rabbit IgG (Santa Cruz Biotechnology) at a 1:4000 dilution for 1 hour. GAPDH, β-actin, and α-tubulin expression levels were used as protein loading controls.

### Cytosol and membrane protein extraction

Cells were lysed in homogenization buffer (20 mM Tris (pH 7.5), 5 mM EGTA, and 20 mM EDTA) containing protease inhibitors and then disrupted by gentle repeated pipetting through a syringe 30 times on ice. After centrifugation at 12000 rpm for 10 minutes at 4°C, the supernatant, which was the cytoplasmic fraction, was transferred to a new microfuge tube, and the cell pellets were extracted for membrane proteins. After the pellet was washed 2 times, it was lysed in cell lysis buffer as described in the western blotting protocol section and further disrupted every 5 minutes for 45 minutes using a vortex mixer. After centrifugation at 12000 rpm for 30 minutes at 4°C, the supernatant was harvested as the membrane fraction. Finally, cytoplasmic and membrane fractions were examined via 10% SDS-PAGE gels.

### Cell-matrix adhesion assays

To prepare coated plates, different concentrations (0, 0.1, 1, or 10 μg/ml) of extracellular matrix (ECM) proteins (fibronectin, collagen I, and collagen IV purchased from Sigma-Aldrich) were evenly incubated in 12-well plates at 37°C for 3 hours. After incubation, the solutions were removed from each well, and the wells were blocked with 0.2% BSA at 37°C for another 1 hour; the plates were washed with PBS 2 times and further dried in an incubator overnight. For the adhesion assay, cells were detached with enzyme-free cell dissociation solution or 21 μM trypsin-EDTA (1X) (Thermo Fisher Scientific), and then 6 × 10^4^ T47D cells were seeded in a 12-well plate for a 2-hour incubation. Unbound cells were removed by gently washing with PBS 2 times, and adherent cells were fixed with 4% formaldehyde and stained with Hoechst 33342 dye. Cells were washed with PBS and imaged using a 4X objective on a Leica DMI 4000B fluorescence microscope (Leica Microsystems). For different experimental observations, cells were treated with different concentrations of nicotine (0, 1, 10, or 100 μM), NNK (0, 10, 100, or 1000 nM), or 10 μM natural compounds (curcumin, calebin A, or 3’ PS).

### Establishment of stable SGSM2 siRNA-expressing cell lines

Scramble (sc) and RNAi (si) sequences for *SGSM2* were designed using OligoEngine 2.0 software. At least two independent RNAi sequences were used to ablate *SGSM2* expression in T47D cells. All sequences were cloned into a pSUPER.retro.neo+gfp vector followed by pSUPER RNAi System Protocols (OligoEngine Co.). T47D cells were transfected with the *SGSM2* pSUPER-si1, si2, and pSUPER-sc vectors by electroporation with a Neon® system (Thermo Fisher Scientific Inc.). To select stable cell lines, cells were treated with 2.5 mg/mL G418, and clones were isolated after 25–30 days in selection medium to ensure G418-resistant clone formation. The plasmids were constructed using the following sequences: *SGSM2* si1 target sequence, 5’-TCCGATGAAAGACGCTGGT-3’ and *SGSM2* si2 target sequence, 5’-CCTGCACCGCATAGACAAG-3’; *SGSM2* scramble target sequence, 5’-GTATGGAGGCACATACTCG-3’.

### Wound-healing migration assay

A culture insert (Ibidi GmbH) was used to generate a 500 ± 50-μm gap and placed in each well of 12-well plates; then, 3.5 × 10^4^ T47D cells stably expressing SGSM2 si1, si2 or scramble vectors were seeded into both sides of each insert. After cell seeding for 24 hours, we removed all the culture inserts, exchanged the medium for new growth medium, and imaged cell migration at 0, 24, and 48 hours. Cells were fixed with 4% formaldehyde and stained with Hoechst 33342 dye. Images of all movement in the wound area were captured with a 10X objective on a Leica DMI 4000B fluorescence microscope.

### Co-immunoprecipitation (Co-IP)

Cells were lysed on ice in RIPA lysis buffer (20 mM Tris-HCL (pH 7.5), 150 mM NaCl, 1 mM Na_2_EDTA, 1 mM EGTA, 1% NP-40, 1% sodium deoxycholate, 2.5 mM sodium pyrophosphate, 1 mM β-glycerophosphate, 1 mM Na_3_VO_4_, 1 μg/ml leupeptin, 0.5 M sodium orthovanadate, and 200 μM PMSF). Cell lysates were further disrupted 3 times over 150 minutes using a vortex mixer and then spun down at 12000 rpm for 30 minutes. Protein lysates containing equal amounts of total protein (~2 mg) were incubated with 2 μg of specific antibodies against E-cadherin and β-catenin overnight at 4°C, and the mixture was further incubated with protein A/G agarose beads (EMD Millipore) for 2 hours at 4°C. Thereafter, the samples were washed four times with PBS and examined with 10% SDS-PAGE gels. In parallel, an equal amount of nonspecific IgG was used as the negative control. In addition, 4 mM EGTA was used to disrupt E-cadherin-mediated cell-cell contacts, and 1.8 mM Ca2^+^ was used to reform adherent junctions; both experiments were performed using a well-established procedure [29].

### Immunofluorescence (IF) staining

Cells were seeded on glass slides for 24 hours and then fixed with 4% paraformaldehyde (Merck) for 5 minutes at room temperature. The fixed cells were rinsed with PBS 3 times and incubated in blocking solution (10% FBS in PBS) for 30 minutes. Cells were then incubated with primary antibodies (anti-SGSM2, anti-E-cadherin, anti-EEA1, anti-p-FAK(Y397), and anti-β-tubulin at 1:200 dilution and Phalloidin-iFluor 488) for 2 hours and further incubated with the specific secondary antibody for 1 hour. Finally, the samples were mounted with Gel Mount (Sigma-Aldrich) and imaged using confocal microscopy (Leica TCS SP5, Leica). Mouse anti-β-tubulin antibody was purchased from GeneTex company (GTX11307). The secondary antibodies were purchased from Jackson ImmunoResearch Lab Co. Phalloidin-iFluor 488 (ab176753, Abcam) or rhodamine phalloidin (# PHDR1, Cytoskeleton Inc.) were used for staining F-actin, which was performed according to the manufacturer’s instructions.

### Fluorescence resonance energy transfer (FRET)

The acceptor bleaching FRET method was used according to the manufacturer’s instructions (FRET Wizards in the Leica Application suite). Briefly, the initial donor (FITC 488 nm) image represents donor fluorescence in the presence of the acceptor rhodamine. After complete photobleaching of the acceptor, a second donor image was collected.

### Statistical analysis

The cell numbers in the cell adhesion and cell migration assays were determined using ImageJ software. All data are presented as the mean±S.E.M. of more than three independently repeated experiments. *SGSM2* mRNA expression in paired normal *vs*. tumour tissues from BC patients was compared with paired t-tests. Independent t-tests (Welch’s t-test for heterogeneous variance) were used to compare the differences between two groups, and one-way ANOVA (LSD, Scheffe, and Tukey HSD test for homogeneity of variance; Dunnett T3 and Games-Howell test for heterogeneous variance) was used to compare differences among three or more groups. A Pearson correlation test was used to analyse the correlation of *SGSM2* and *CDH1* with the expression of various genes; a partial correlation test was used to verify the association among *ERBB2, SGSM2*, and *CDH1* expression in normal tissue samples. All statistical comparisons were performed using Sigma Plot graphing software and Statistical Package for the Social Sciences v.11.0.0 (SPSS). A P-value of 0.05 or less indicated statistical significance.

## Abbreviations

SGSM: small G protein signalling modulator
PCR: polymerase chain reaction
RT-PCR: reverse transcription PCR
EGTA: ethylene glycol tetraacetic acid
BC: breast cancer
EMT: epithelial-mesenchymal transition
ER: oestrogen receptor
FGF: fibroblast growth factor
TGF-β: transforming growth factor β
EGF: epidermal growth factor
IGF1: insulin-like growth factor-1
TNFα: tumour necrosis factor-α
MMPs: metalloproteinases
HGF: hepatocyte growth factor
SF: scatter factor
Rap: Ras-associated protein
RAPID: RAP-interacting domain
GAP: GTPase-activating protein
FNBP1L: formin-binding protein 1-like
WASL: Wiskott-Aldrich syndrome-like
CDC42: cell division cycle 42
CDH1: cadherin 1
GUS: β-glucuronidase
HER2: human epidermal growth factor receptor 2
IHC: immunohistochemistry
GAPDH: glyceraldehyde 3-phosphate dehydrogenase
ECM: extracellular matrix
FRET: fluorescence resonance energy transfer
FITC: fluorescein isothiocyanate
PR: progesterone receptor
IP: immunoprecipitation
FBS: foetal bovine serum
E2: 17β-estradiol
LN: lobular neoplasia
ILC: invasive lobular carcinoma
IDC: invasive ductal carcinoma
FAK: focal adhesion kinase
