# Supplementary material for: Small G protein signalling modulator 2 (SGSM2) is involved in oestrogen receptor-positive breast cancer metastasis through enhancement of migratory cell adhesion via interaction with E-cadherin
Source: Cell Adh Migr. 2019 Feb 11;13(1):120–37. doi: 10.1080/19336918.2019.1568139 (PMC6527379; doi:10.1080/19336918.2019.1568139)
Supplement: Supplemental Material [file kcam-13-01-1568139-s001.docx]

**Small G protein signalling modulator 2 (SGSM2) is involved in oestrogen receptor-positive breast cancer metastasis through enhancement of migratory cell adhesion via interaction with E-cadherin**

*Juo-Han Lin^1^, Wen-Jui Lee^2^, Han-Chung Wu^3^, Chih-Hsiung Wu^4,5^, Li-Ching Chen^6,7,8^, Chi-Cheng Huang^4,9,10^,Hang-Lung Chang^5^, Tzu-Chun Cheng^11^, Hui-Wen Chang^12^, Chi-Tang Ho^13^, Shih-Hsin Tu^4,6,7*^, Yuan-Soon Ho^8,11,12,14*^*

^1^Ph.D. Program for Cancer Molecular Biology and Drug Discovery, College of Medical Science and Technology, Taipei Medical University and Academia Sinica, Taipei, Taiwan

^2^Ph.D. Program for Neural Regenerative Medicine, College of Medical Science and

Technology, Taipei Medical University and National Health Research Institutes, Taiwan

^3^Institute of Cellular and Organismic Biology, Academia Sinica, Taipei, Taiwan

^4^Department of Surgery, School of Medicine, College of Medicine, Taipei Medical University, Taipei, Taiwan

^5^Department of General Surgery, En Chu Kong Hospital, New Taipei City, Taiwan

^6^Breast Medical Center, Taipei Medical University Hospital, Taipei, Taiwan

^7^Taipei Cancer Center, Taipei Medical University, Taipei, Taiwan

^8^TMU Research Center of Cancer Translational Medicine, Taipei Medical University, Taipei, Taiwan

^9^School of Medicine, College of Medicine, Fu-Jen Catholic University, New Taipei City, Taiwan

^10^Department of Surgery, Fu-Jen Catholic University Hospital, New Taipei City, Taiwan

^11^Graduate Institute of Medical Sciences, College of Medicine, Taipei Medical University, Taipei, Taiwan

^12^Department of Laboratory Medicine, Taipei Medical University Hospital, Taipei, Taiwan

^13^Department of Food Science, Rutgers University, New Brunswick, New Jersey 08901, USA

^14^School of Medical Laboratory Science and Biotechnology, College of Medical Science and Technology, Taipei Medical University, Taipei, Taiwan

**Supplementary materials**

3 supplementary tables, 11 supplementary figures, and 8 supplementary references

**Supplementary Table S1** *SGSM2* expression was detected by RNAseq in tumour tissues of TCGA database.

| Factors | SGSM2  RNAseq  (Mean ± S.E.M) | Statistics  P-value | Case Number | Factors | SGSM2  RNAseq  (Mean ± S.E.M) | Statistics  P-value | Case Number |
| --- | --- | --- | --- | --- | --- | --- | --- |
| **PAM50** | ANOVA ** P<0.001** | | Total=849 | **Stage** | ANOVA P=0.451 | | Total=1094 |
| Luminal A | 10.12 ± 0.03 | Scheffe | 422 | stage I  stage II  stage III  stage IV  Stage X | 10.22 ± 0.06 |  | 182 |
| Luminal B | 9.94 ± 0.05 | 0.052 | 194 |  | 10.10 ± 0.03 |  | 626 |
| HER2-enriched | 9.46 ± 0.08 | ** <0.001** | 67 |  | 10.12 ± 0.05 |  | 252 |
| Basal-like | 9.58 ± 0.06 | ** <0.001** | 142 |  | 10.28 ± 0.16 |  | 20 |
| Normal-like | 10.17 ± 0.11 | 0.998 | 24 |  | 10.16 ± 0.19 |  | 14 |
| **ER status** | Student’s t-test ** P<0.001** | | Total=780 | **T status** | ANOVA ** P= 0.005** | | Total=1102 |
| Negative | 9.55 ± 0.05 |  | 179 | T1  T2 | 10.23 ± 0.05 | Tukey HSD | 281 |
| Positive | 10.03 ± 0.03 |  | 601 |  | 10.06 ± 0.03 | ** 0.028** | 640 |
| **PR status** | Student’s t-test ** P<0.001** | | Total=777 | T3 | 10.25 ± 0.07 | 0.997 | 138 |
| Negative | 9.74 ± 0.05 |  | 255 | T4 | 9.98 ± 0.12 | 0.332 | 40 |
| Positive | 10.02 ± 0.03 |  | 522 | TX | 10.59 ± 0.34 | 0.937 | 3 |
| **HER2 status** | ANOVA ** P<0.001** | | Total=909 | **N status** | ANOVA P=0.075 | | Total=1102 |
| Negative | 10.15 ± 0.03 | Scheffe | 565 | N0  N1 | 10.13 ± 0.04 | 0.267 | 516 |
| Positive | 9.80 ± 0.06 | ** <0.001** | 164 |  | 10.13 ± 0.04 | 0.324 | 367 |
| Equivocal | 10.26 ± 0.06 |  | 180 | N2 | 9.97 ± 0.07 | Tukey HSD | 120 |
| **New_neoplasm** | ANOVA P=0.311 | | Total=102 | N3 | 10.29 ± 0.09 | ** 0.04** | 79 |
| Distant metastasis | 10.08 ± 0.09 | ** P=0.031** | 62 | NX | 10.23 ± 0.21 | 0.666 | 20 |
| Locoregional disease | 9.49 ± 0.35 | LSD | 9 | **M status** | ANOVA ** P<0.001** | | Total=1102 |
| Locoregional recurrence | 10.19 ± 0.25 | *** *P=0.037** | 12 | M0, cM0(i+)  M1  MX | 10.06 ± 0.03 | Scheffe | 917 |
| New primary tumor | 9.96 ± 0.15 |  | 19 |  | 10.20 ± 0.17 | 0.714 | 22 |
|  |  |  |  |  | 10.46 ± 0.07 | ** <0.001** | 163 |

The RNAseq data of the TCGA Breast Cancer (BRCA) cohort was from the UCSC Xena browser ([http://xena.ucsc.edu](http://xena.ucsc.edu/)). Gene expression is displayed as log2 (norm_count +1) values. Independent t-tests (Welch’s t-test for heterogeneous variance) were used to compare differences between two groups, and one-way ANOVA (LSD, Scheffe, and Tukey HSD test for homogeneity of variance; Dunnett T3 and Games-Howell test for heterogeneous variance) was used to compare differences among three or more groups. Quantitative data are presented as the mean ± S.E.M; the mean is the average log2 (SGSM2 norm_count +1) value. All P-values are two-tailed, and  indicates a P-value<0.05;  P-value<0.01, and  P-value<0.001. **Bold text** indicates the significance of the analysis.

**Supplementary Table S2** The correlation between *SGSM2* and the expression of various genes in the TCGA Breast Cancer (BRCA) cohort.





The RNAseq data of the TCGA Breast Cancer (BRCA) cohort was from the UCSC Xena browser ([http://xena.ucsc.edu](http://xena.ucsc.edu/)). A Pearson correlation test was used to analyse the correlation of *SGSM2* and *CDH1* with the expression of various genes; a partial correlation test was used to verify the association with *ERBB2*, *SGSM2*, and *CDH1* expression in normal tissue samples. The correlation values are presented in ();  indicates P-value<0.05;  P-value<0.01, and  P-value<0.001. “n” indicates the number of cases. Red text indicates a positive correlation, and green text indicates a negative correlation.

**Supplementary Table S3** The RNAseq values of multiple genes analysed in the TCGA Breast Cancer (BRCA) cohort.

| **Gene** | Paired normal tissues  RNAseq  (Mean ± S.E.M)  n=114 | Paired tumour tissues  RNAseq  (Mean ± S.E.M)  n=114 | All tumour tissues  RNAseq  (Mean ± S.E.M)  n=1104 | **Gene** | Paired normal tissues RNAseq  (Mean ± S.E.M)  n=114 | Paired tumour tissues  RNAseq  (Mean ± S.E.M)  n=114 | All tumour tissues  RNAseq  (Mean ± S.E.M)  n=1104 |
| --- | --- | --- | --- | --- | --- | --- | --- |
| **SGSM2** | 10.53 ± 0.63 | 9.75 ± 0.74 | 10.12 ± 0.80 | **TWIST1** | 8.17 ± 1.16 | 6.71 ± 1.27 | 6.71 ± 1.39 |
| **CDH1** | 11.91 ± 3.00 | 13.26 ± 3.00 | 12.80 ± 1.68 | **VIMENTIN** | 15.56 ± 0.87 | 14.15 ± 0.87 | 14.17 ± 0.93 |
| **ESR1** | 11.31 ± 1.23 | 12.04 ± 2.76 | 11.67 ± 3.19 | **CTNNB1** | 13.55 ± 0.48 | 13.33 ± 0.49 | 13.15 ± 0.61 |
| **ESR2** | 4.82 ± 1.28 | 2.84 ± 0.94 | 2.98 ± 0.91 | **TP53** | 10.49 ± 0.58 | 10.60 ± 0.71 | 10.51 ± 0.86 |
| **ERBB2** | 11.86 ± 1.20 | 12.88 ± 1.50 | 12.97 ± 1.49 | **SRC** | 9.40 ± 0.64 | 9.99 ± 0.75 | 10.17 ± 0.73 |
| **GRB7** | 8.03 ± 2.34 | 9.05 ± 1.33 | 9.26 ± 1.44 | **HRAS** | 8.34 ± 0.74 | 8.59 ± 0.78 | 9.00 ± 0.92 |
| **EGFR** | 10.67 ± 0.79 | 7.83 ± 1.82 | 7.34 ± 2.03 | **KRAS** | 9.77 ± 0.37 | 10.50 ± 0.52 | 10.26 ± 0.63 |
| **EGF** | 7.94 ± 2.62 | 6.55 ± 2.33 | 6.30 ± 2.24 | **PIK3A** | 9.76 ± 0.60 | 9.26 ± 0.75 | 8.98 ± 0.76 |
| **FGFR3** | 6.33 ± 1.21 | 7.60 ± 1.99 | 7.98 ± 2.14 | **BRAF** | 7.67 ± 0.54 | 7.64 ± 0.65 | 7.48 ± 0.73 |
| **FGFR2** | 10.03 ± 1.33 | 9.43 ± 1.49 | 9.21 ± 1.67 | **RB1** | 10.39 ± 0.35 | 10.22 ± 0.75 | 9.97 ± 0.85 |
| **FGF1** | 10.15 ± 0.73 | 7.85 ± 1.02 | 7.64 ± 1.28 | **BRCA1** | 7.62 ± 0.49 | 8.56 ± 1.00 | 8.40 ± 1.07 |
| **FGF2** | 10.34 ± 0.94 | 7.05 ± 1.63 | 6.77 ± 1.71 | **BRCA2** | 5.83 ± 1.00 | 7.45 ± 1.15 | 7.12 ± 1.23 |
| **CDH2** | 4.61 ± 1.38 | 6.28 ± 1.57 | 6.25 ± 1.79 | **MKI67** | 7.45 ± 1.39 | 10.73 ± 1.15 | 10.65 ± 1.30 |
| **SNAI1** | 6.76 ± 1.00 | 6.71 ± 0.81 | 6.70 ± 1.06 |  |  |  |  |

Quantitative data are presented as the mean ± S.E.M; the mean is the average log2 (norm_count +1) value. “n” indicates the number of cases.


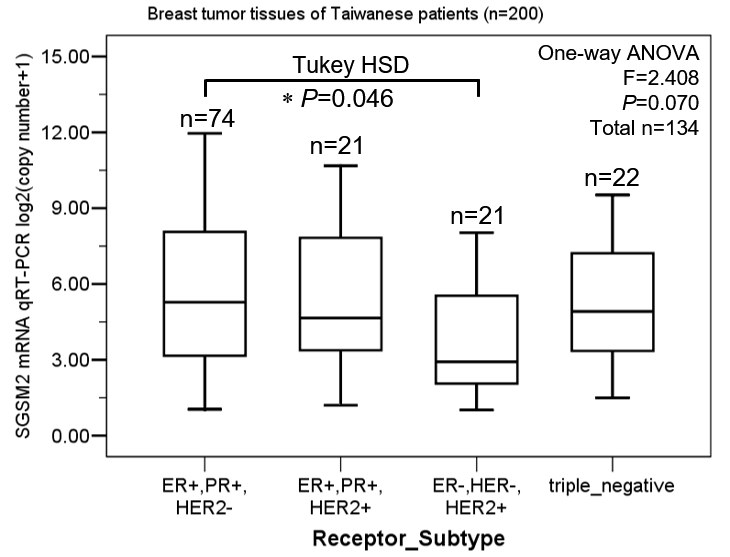

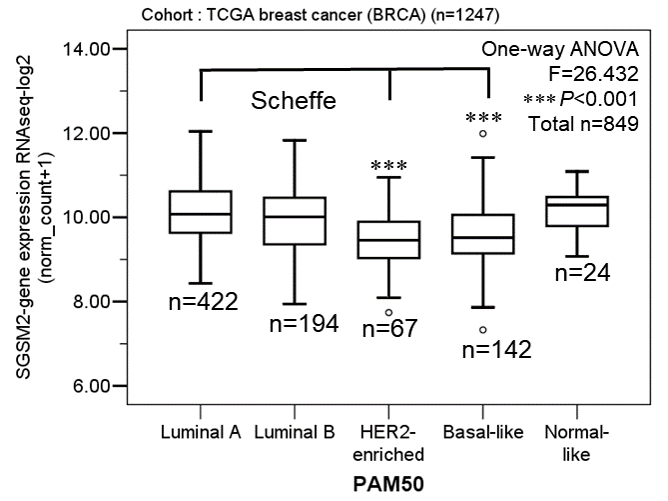

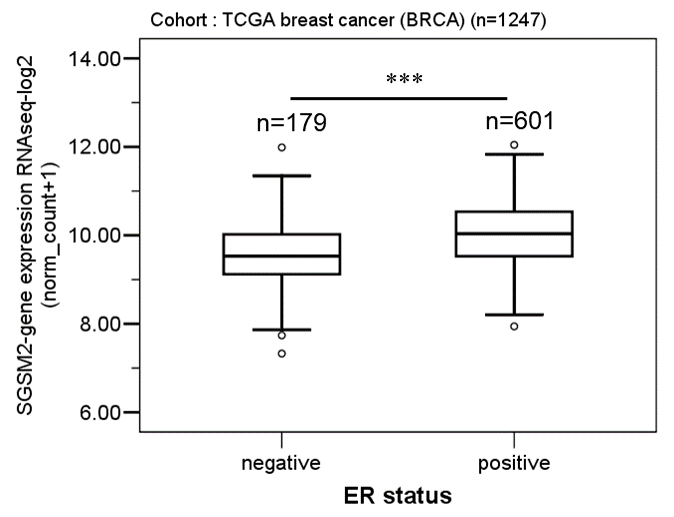


**A**

**B**

**C**

**D**


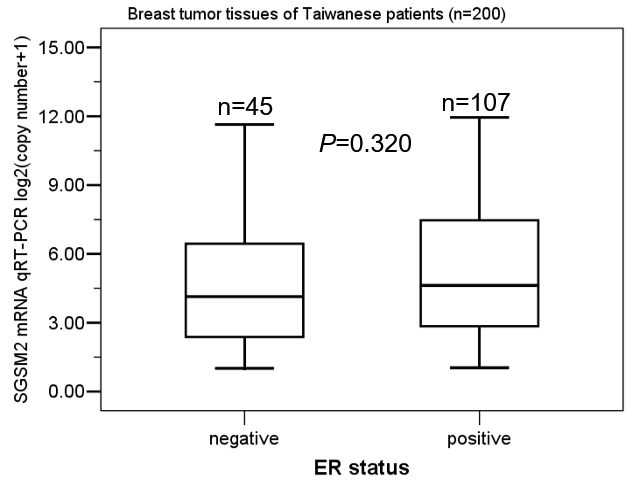


**Supplementary Figure S1. *SGSM2* gene expression in breast tumours according to receptor-defined subtype and PAM50 subtype.** *SGSM2* expression was analysed according to ER status with (A) real-time PCR in Taiwanese patients, (B) RNAseq of the TCGA Breast Cancer (BRCA) cohort using UCSC Xena browser ([http://xena.ucsc.edu](http://xena.ucsc.edu/)). (C) *SGSM2* expression in Taiwanese patients was analysed by receptor-defined subtype. The expression of (D) *SGSM2* was determined according to PAM50 gene expression subtype in the TCGA Breast Cancer (BRCA) cohort. All P-values are two-tailed, and  indicates a P-value<0.05 and  P-value<0.001. “n” indicates the number of cases.


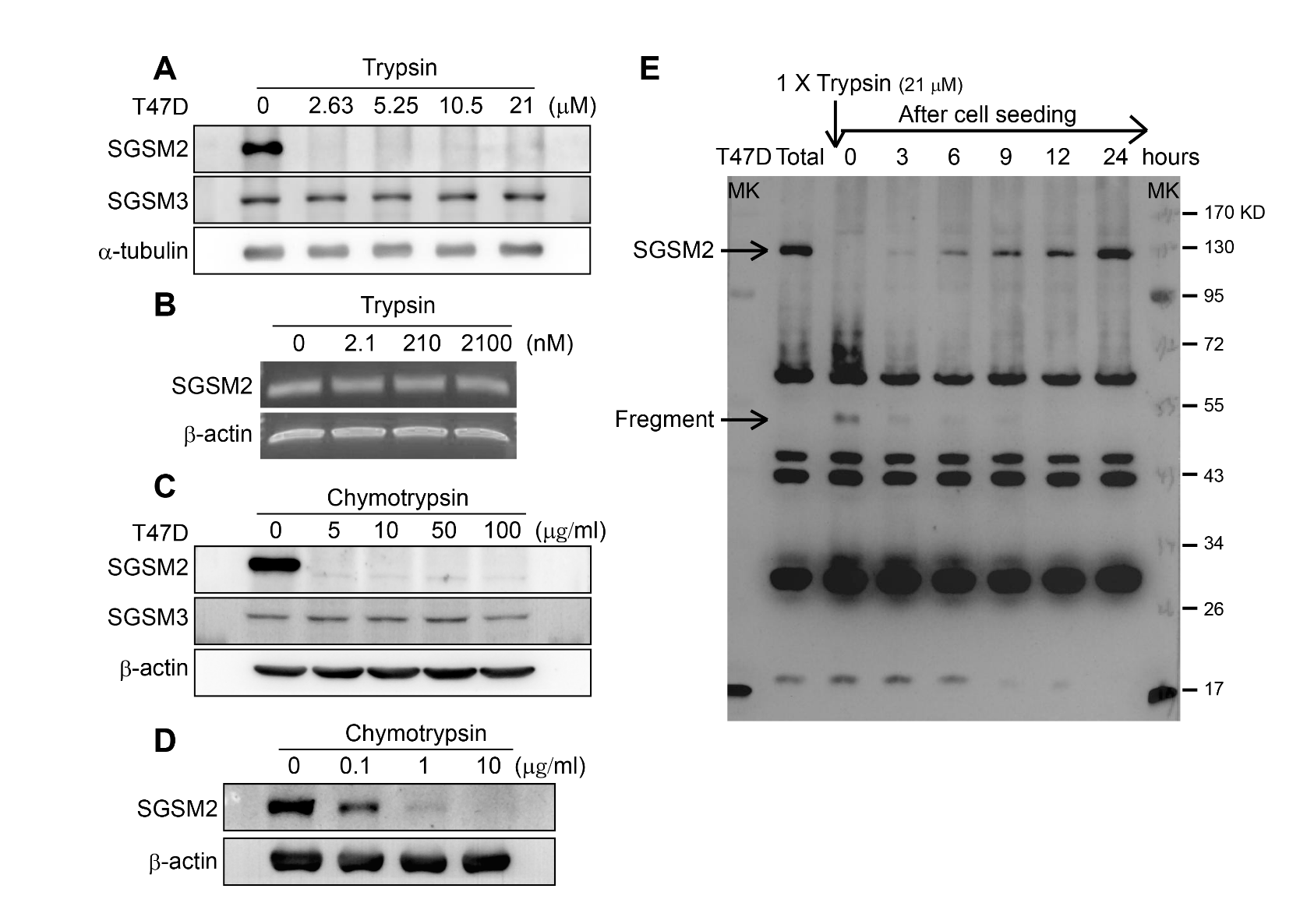


**A**

**E**

**B**

**C**

**D**

**Supplementary Figure S2. Formation of SGSM2 protein fragments was observed after trypsin-EDTA treatment in T47D cells.** (A) To determine SGSM2 and SGSM3 protein levels, T47D cells were treated with different concentrations (0, 2.63, 5.25, 10.5, 21 μM) of trypsin-EDTA for 3 minutes. α-Tubulin was the internal control for western blotting. (B) SGSM2 mRNA levels were detected after treatment with different concentrations (0, 2.1, 210, 2100 nM) of trypsin-EDTA. (C) (D) SGSM2 protein expression was analysed after treatment with different concentrations (0, 1, 5, 10, 50, 100 μg/ml) of chymotrypsin. β-actin was the internal control for western blotting. (E) T47D cells were treated with 1-fold trypsin-EDTA (1X; 21 μM) for subculture, and then, SGSM2 protein was observed at different time points (0, 3, 6, 9, 12, 24 hours) after cell seeding. After trypsin-EDTA treatment, fragments of SGSM2 protein were found near the 55-kDa position. MK indicates the protein marker.


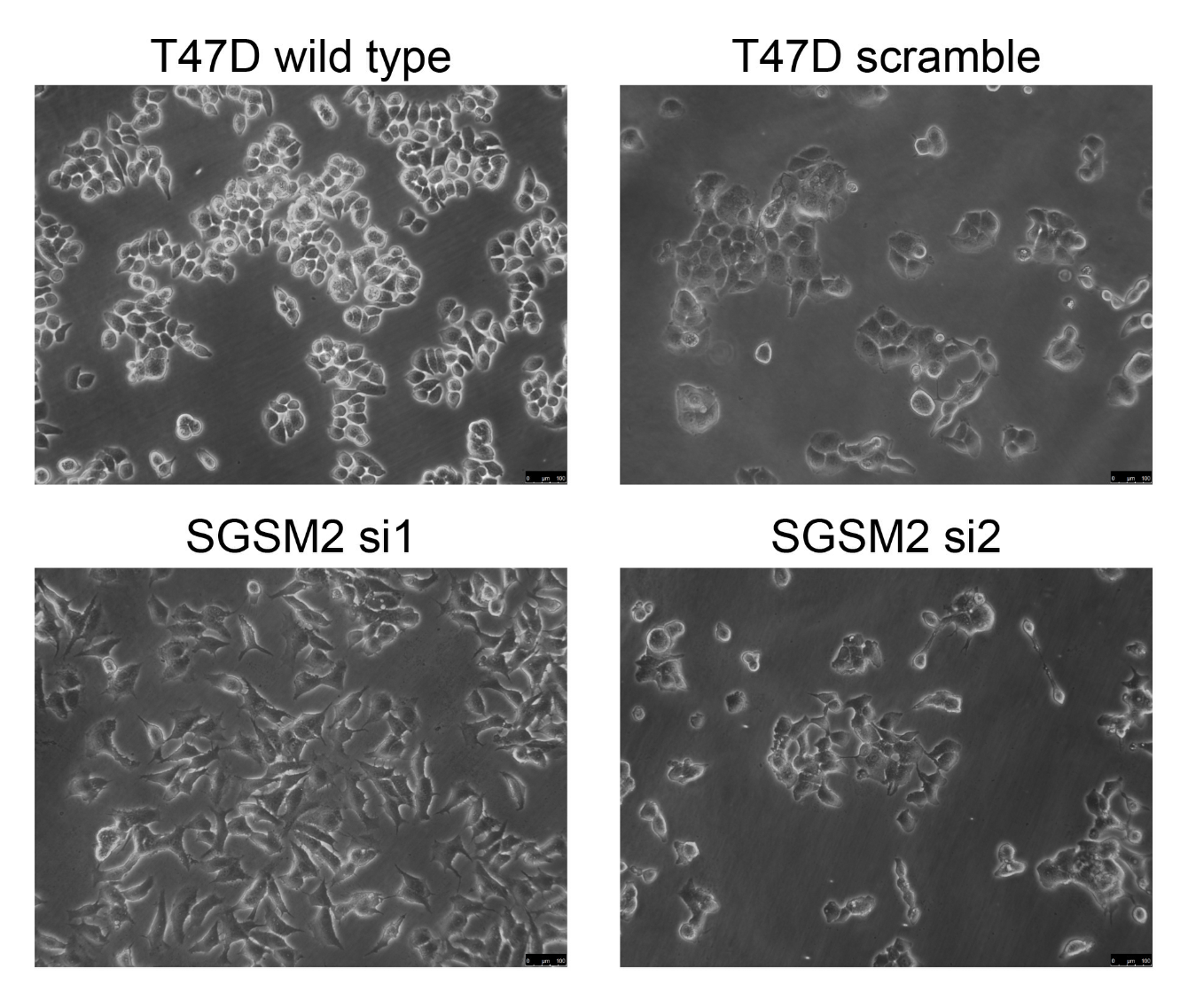


**C**

**A**

**D**

**B**

**Supplementary Figure S3. Brightfield microscope images illustrated cell morphology upon SGSM2 silencing in T47D cells.** Cell shapes of T47D (A) wild-type, (B) SGSM2 scramble, (C) SGSM2 si1, and (D) SGSM2 si2 are displayed. Scale bar, 100 μm.


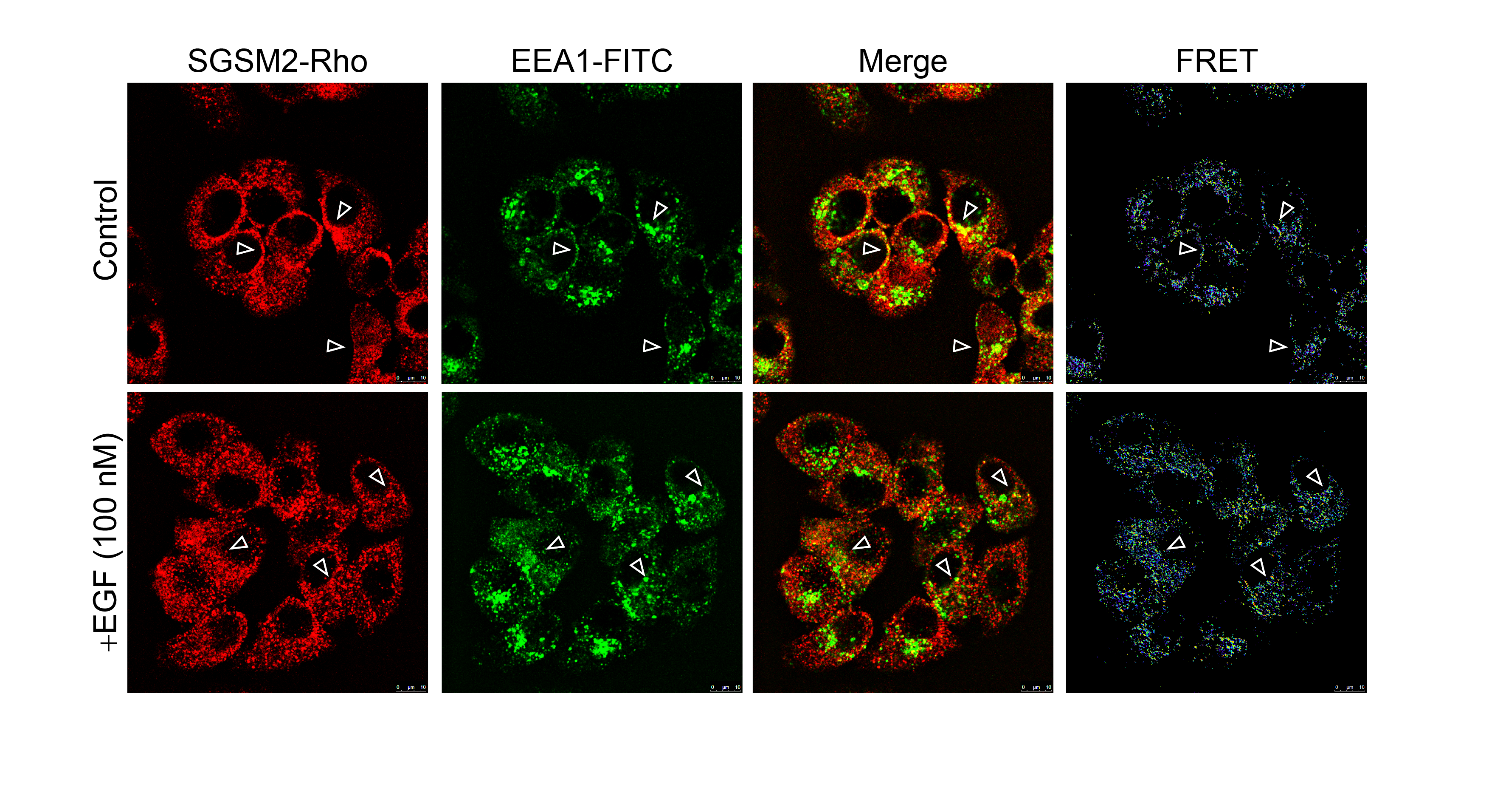


# Supplementary Figure S4. The colocalization of SGSM2-rhodamine (red) and EEA1 (Early endosome antigen 1)-FITC (green) was verified by [immunofluorescence](https://en.wikipedia.org/wiki/Immunofluorescence) staining assays. EGF (100 nM for 60 minutes) was used to induce cell endocytosis. Scale bar, 10 μm. White arrows indicate the colocalization of SGSM2 and EEA1.


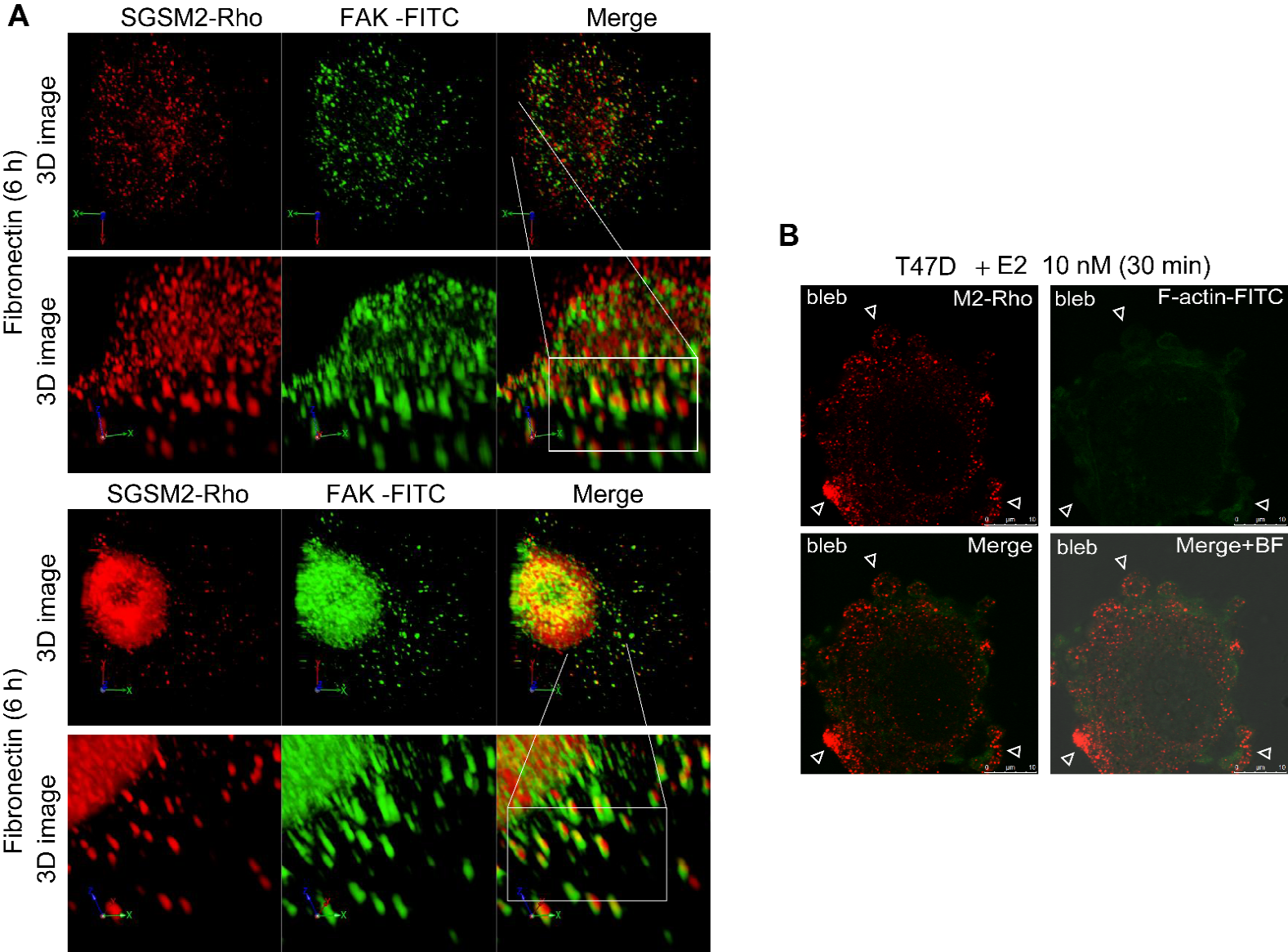


**A**

**B**

**Supplementary Figure S5. IF staining of SGSM2, FAK, and F-actin.** (A) 3D images were obtained with a GE Healthcare DeltaVision Personal Deconvolution Microscope system. SGSM2 and FAK were colocalized at focal adhesions of fibronectin-stimulated T47D cells at the 6-hour mark. SGSM2 is shown in red (rhodamine), and FAK is shown in green (FITC). The white square indicates colocalization of SGSM2 and FAK at focal adhesion sites. (B) Cell bleb formation occurred at the 30-minute mark after E2 (10 nM) exposure. SGSM2 is shown in red (rhodamine), and F-actin is shown in green (FITC). White arrows indicate cell bleb formation. Scale bar, 10 μm.


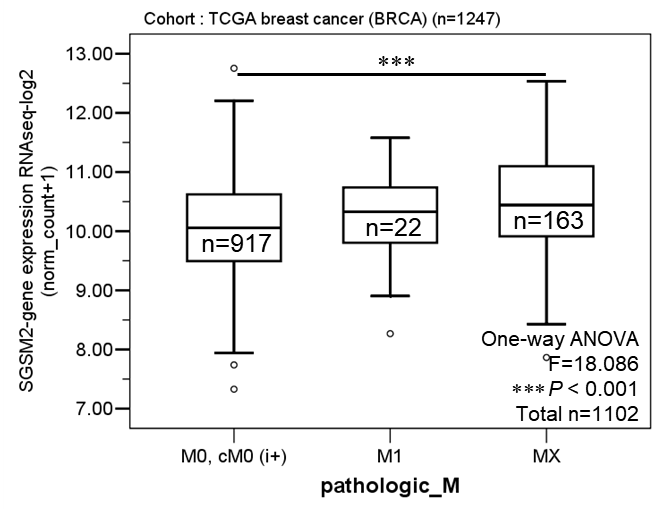

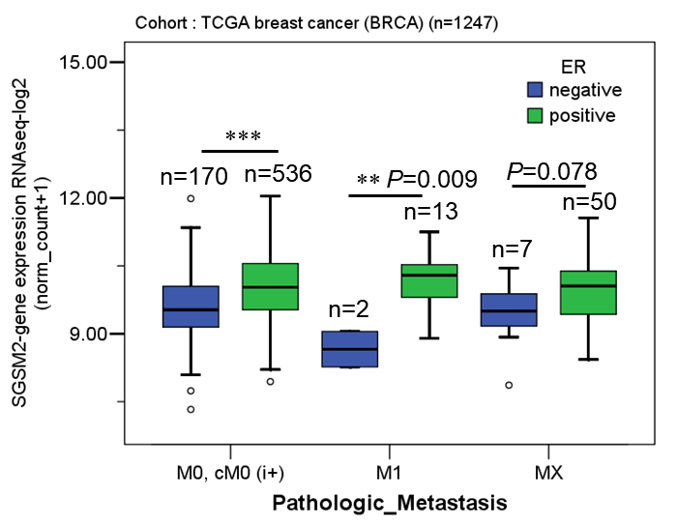


**A**

**B**

**C**

**D**


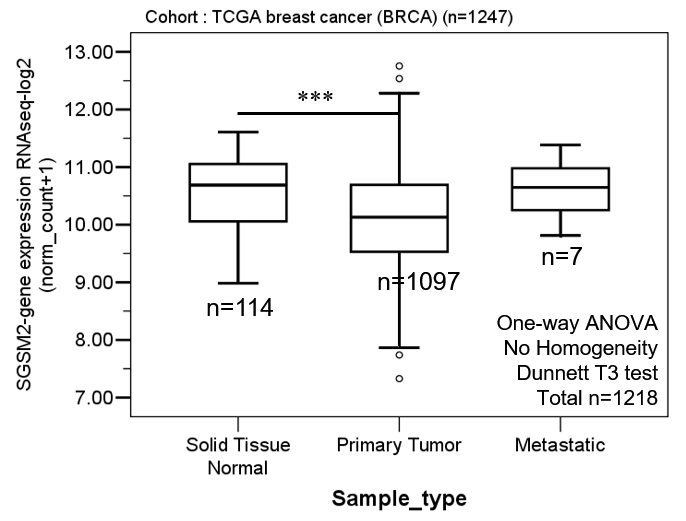

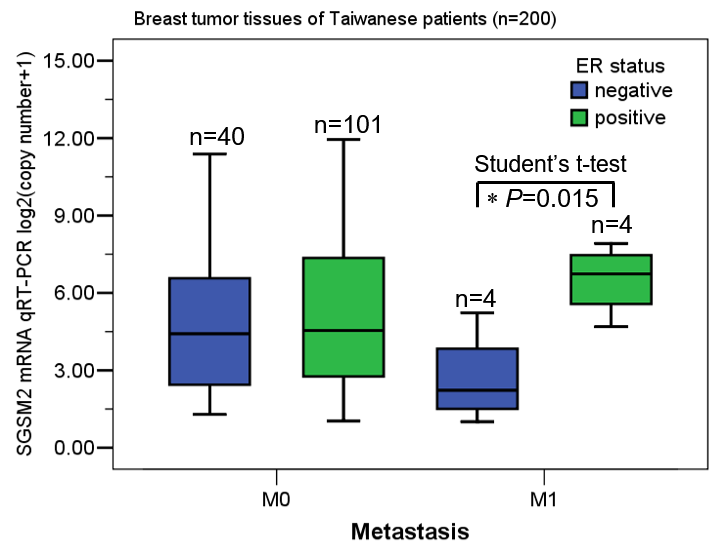


**Supplementary Figure S6. Analysis of the correlation between SGSM2 expression and tumour metastasis.** *SGSM2* expression was analysed according to sample site in (A) the TCGA Breast Cancer (BRCA) cohort using UCSC Xena browser ([http://xena.ucsc.edu](http://xena.ucsc.edu/)). (B) *SGSM2* expression was determined according to pathologic metastasis. SGSM2 was more highly expressed in ER-positive than in ER-negative metastatic tumour patients in the (C) TCGA Breast Cancer (BRCA) cohort and (D) in Taiwanese patients. All P-values are two-tailed, and  indicates a P-value <0.05;  P-value<0.01, and  P-value<0.001. “n” indicates the number of cases.


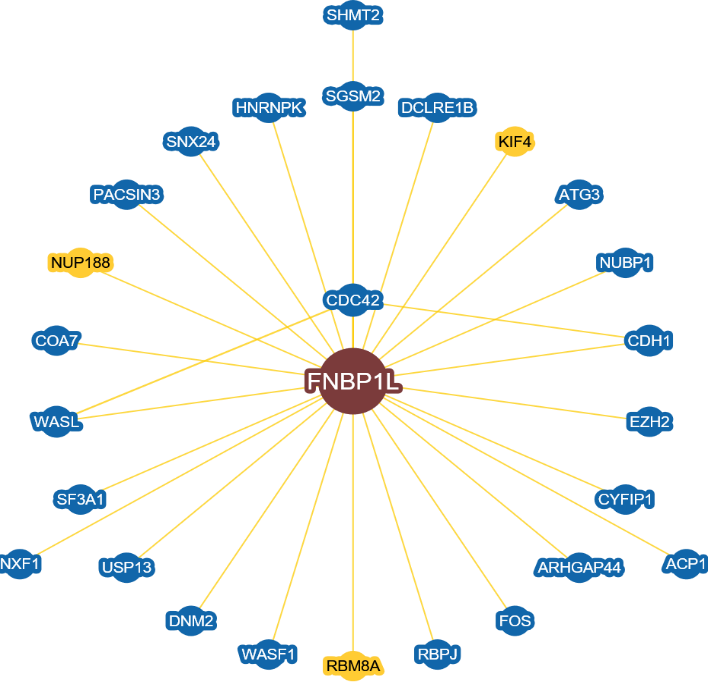

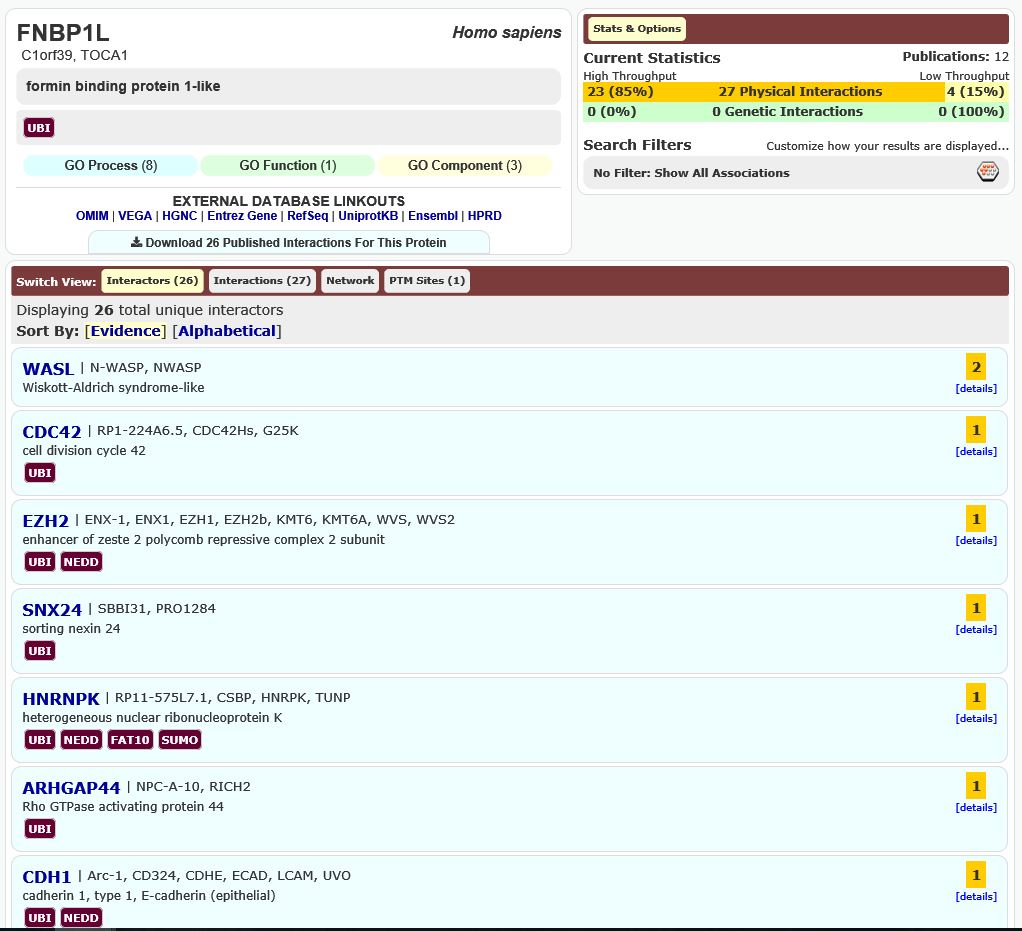

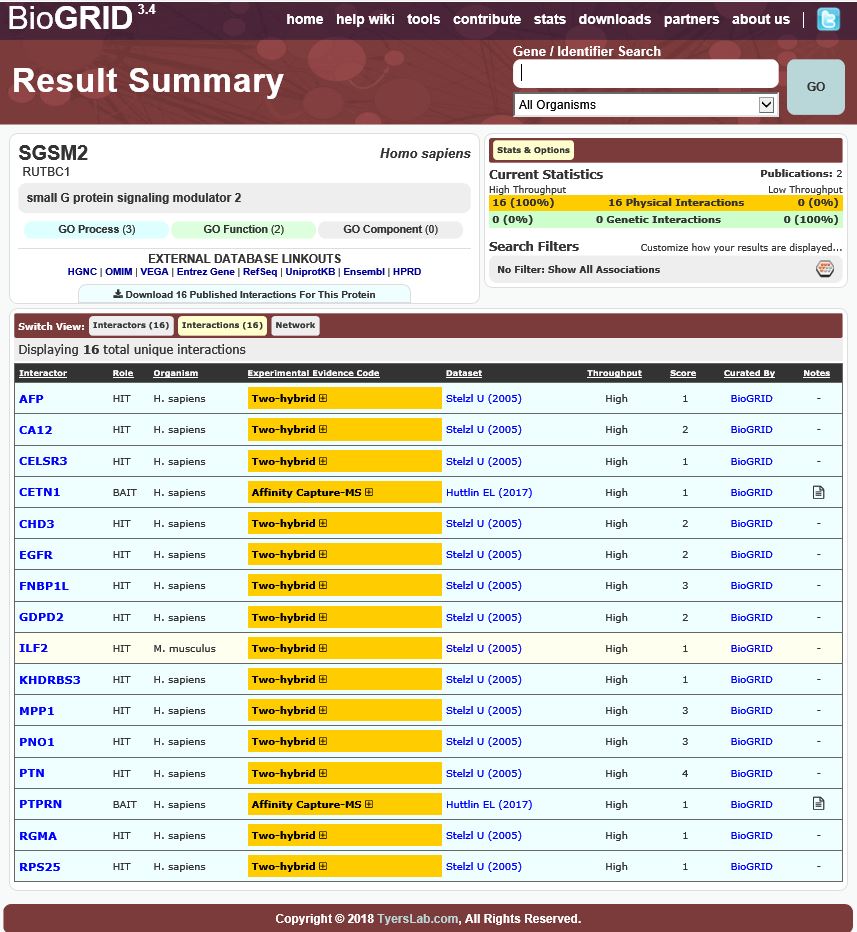

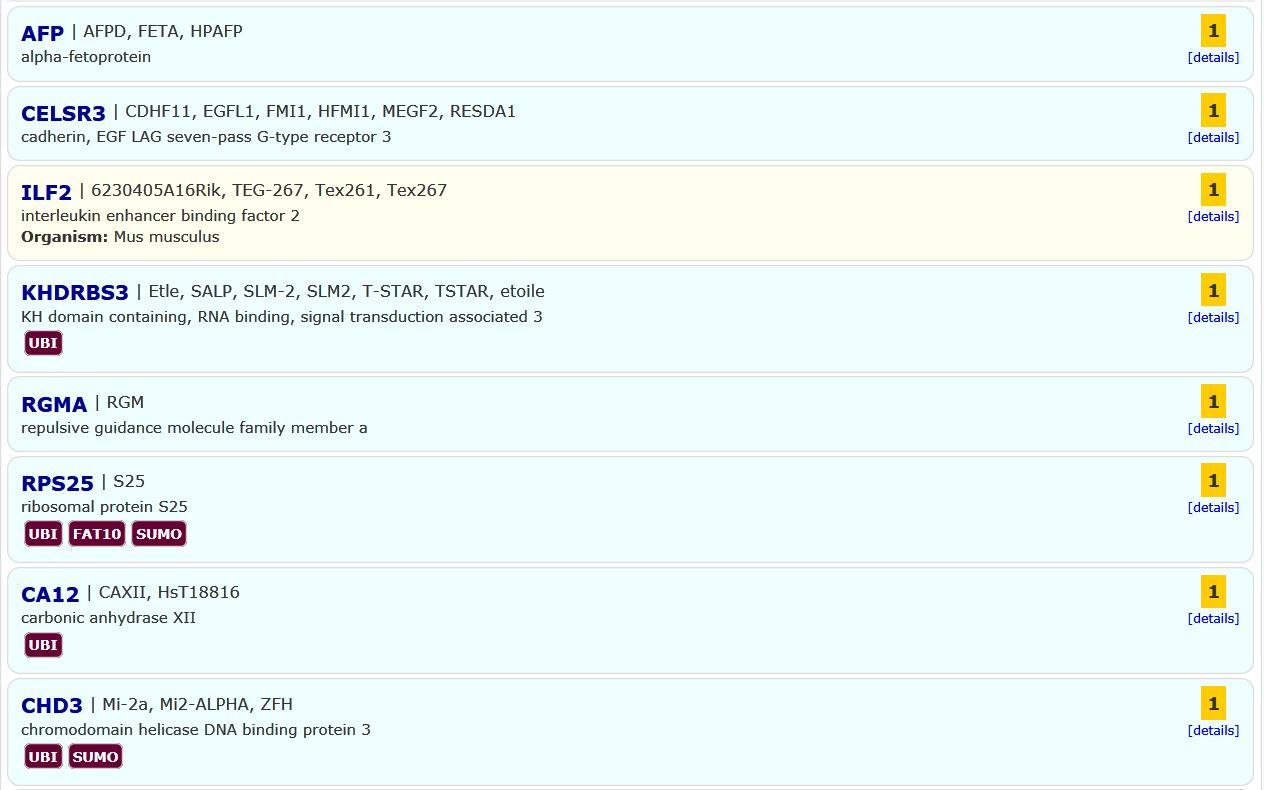

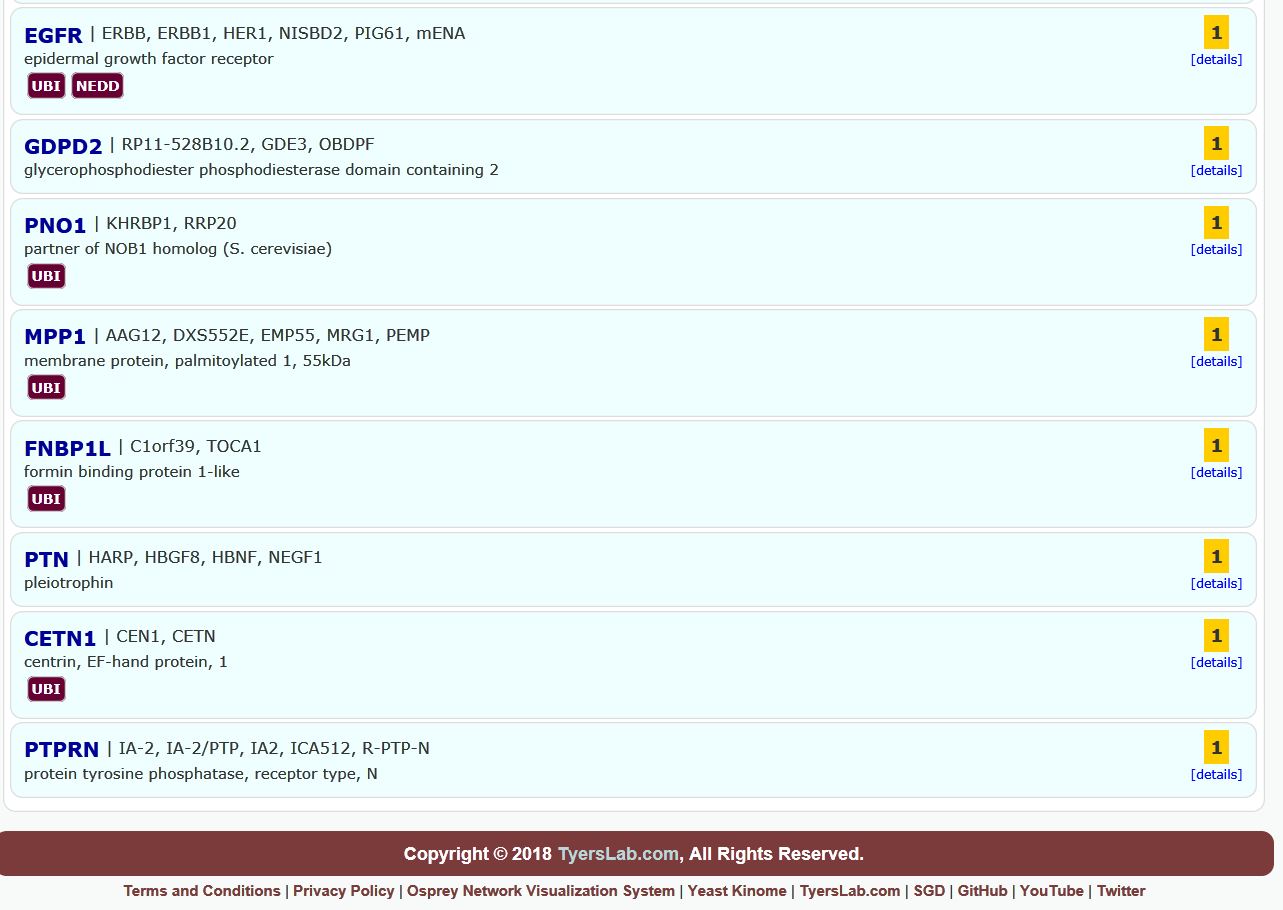

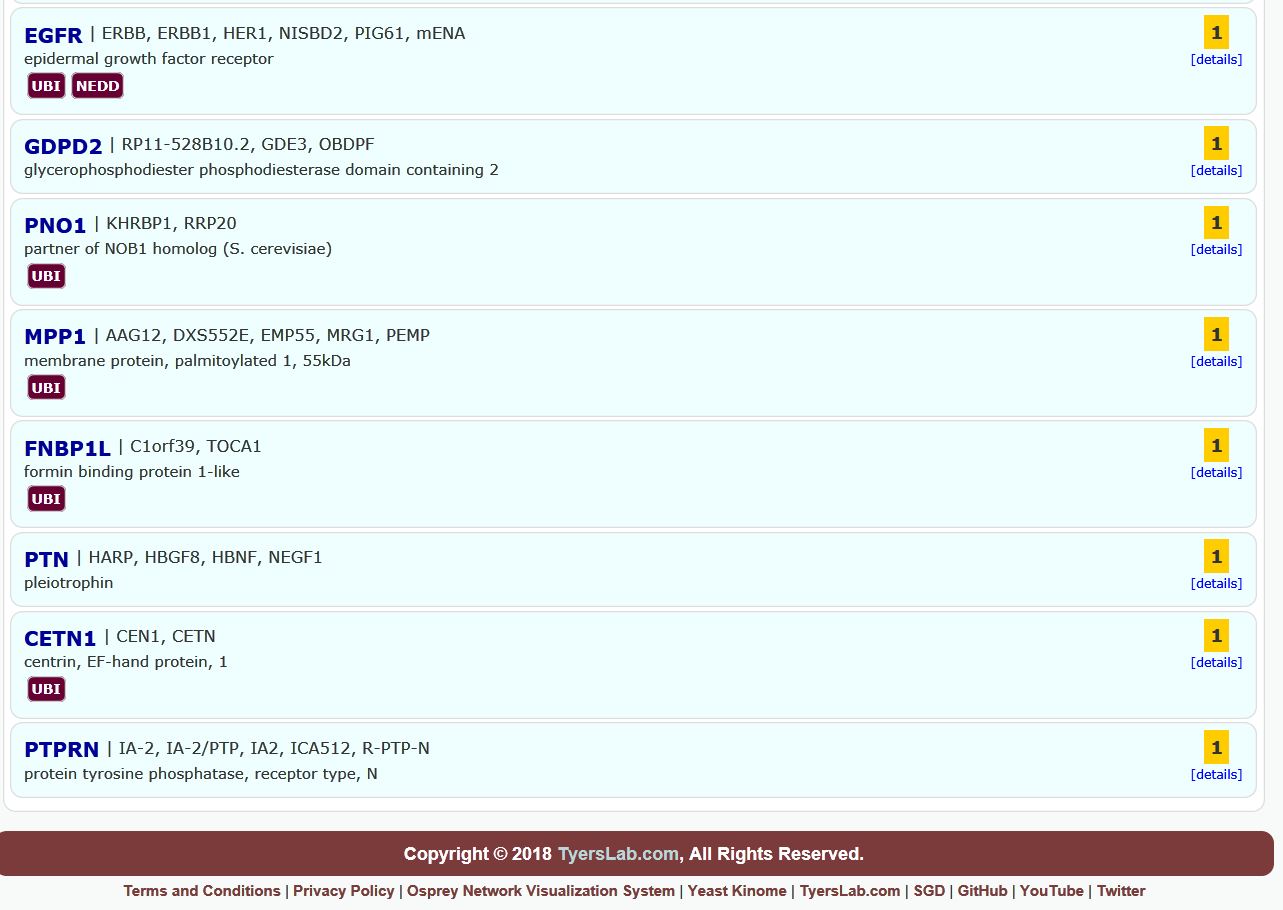

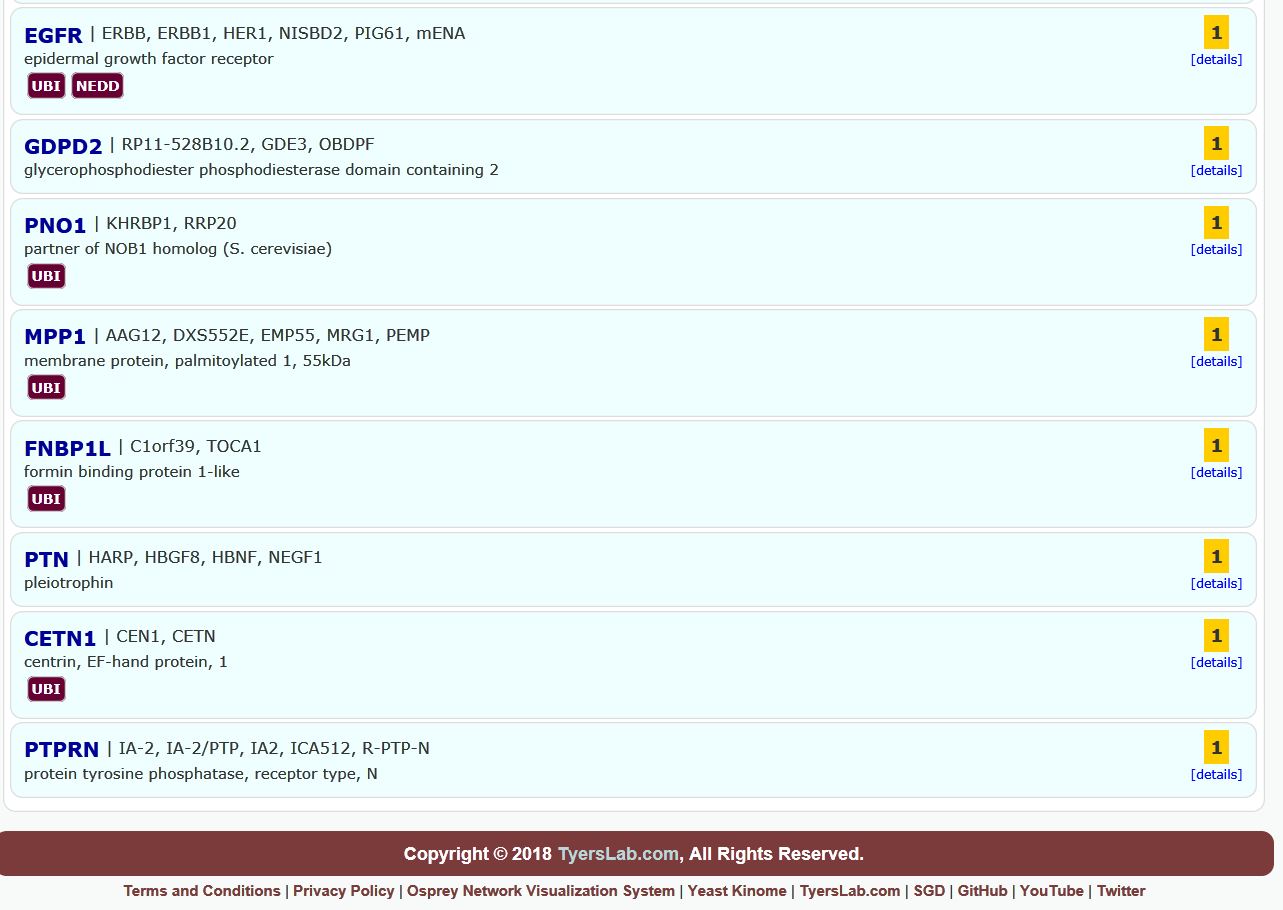


**A**

**B**

**C**

**D**


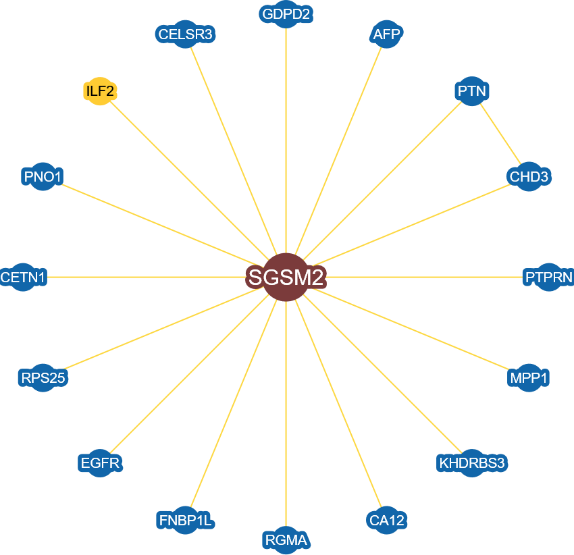


**Supplementary Figure S7. Prediction of a physical interaction between SGSM2 and other proteins using the BioGRID database** (<https://thebiogrid.org/>) ^1^. (A) Sixteen identified proteins with potential physical interactions with SGSM2 are shown in the left panel **^2, 3^**. Four of the interacting proteins (CELSR3, EGFR, FNBP1L, and MMP1) were associated with cell migration and invasion processing (right panel). (B) Three of the potential interacting proteins (WASL, CDC42, and CDH1) are involved in cytoskeleton remodelling and cell-cell junction and can interact with formin-binding protein 1-like (FNBP1L) protein ^4-7^. (C)(D) The networks of all physical interactions with SGSM2 and FNBP1L are displayed. Red circles and red lines indicate the correlation of SGSM2, FNBP1L, CDH1, WASL, and CDC42 proteins.


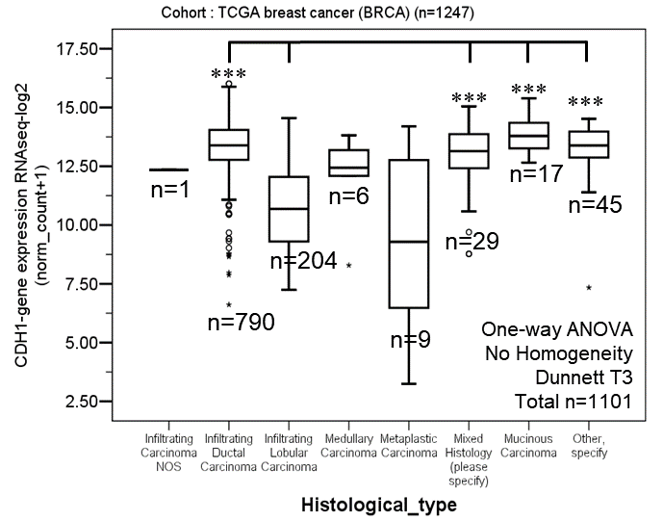


**A**

**B**


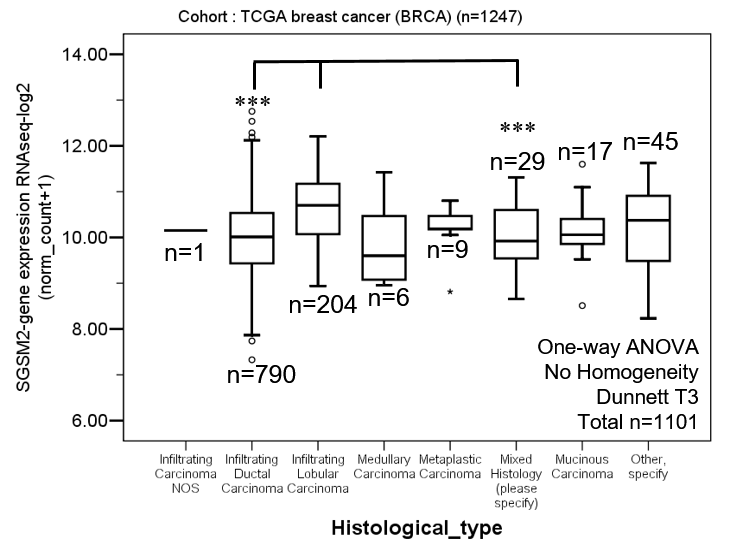


**Supplementary Figure S8. *SGSM2* and *CDH1* gene expression in breast tumour tissues by histological type.** (A) *SGSM2* expression and (B) *CDH1* expression were analysed according to histological type by RNAseq of the TCGA Breast Cancer (BRCA) cohort using the UCSC Xena browser ([http://xena.ucsc.edu](http://xena.ucsc.edu/)). All P-values are two-tailed, and  indicates a P-value<0.05;  and  P-value<0.001. “n” indicates the number of cases.


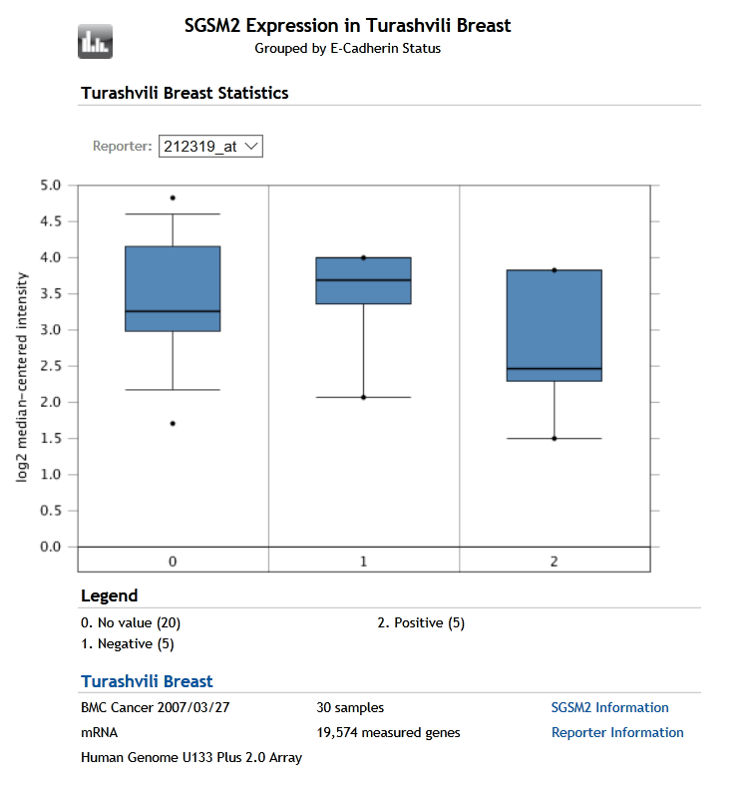

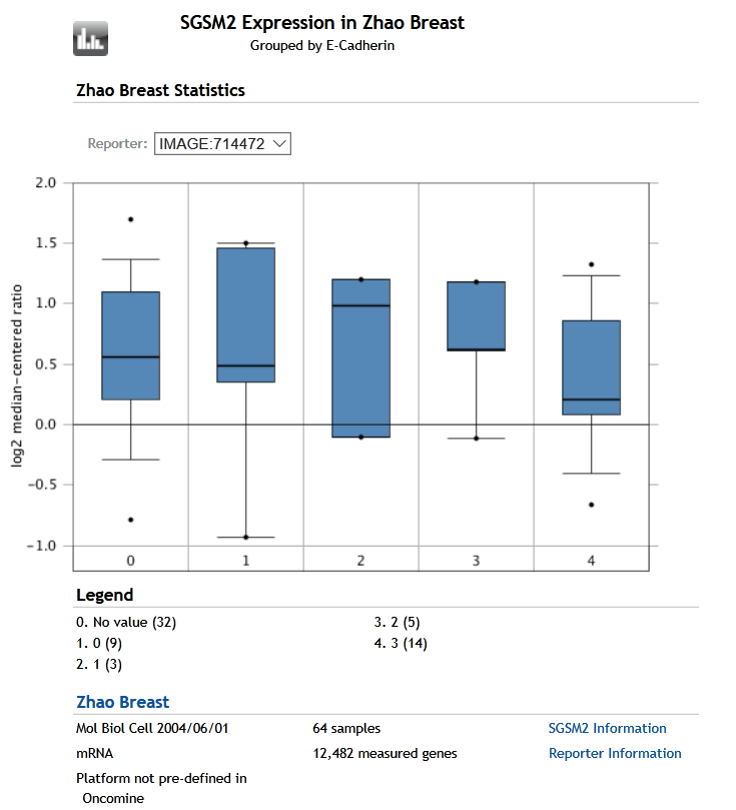

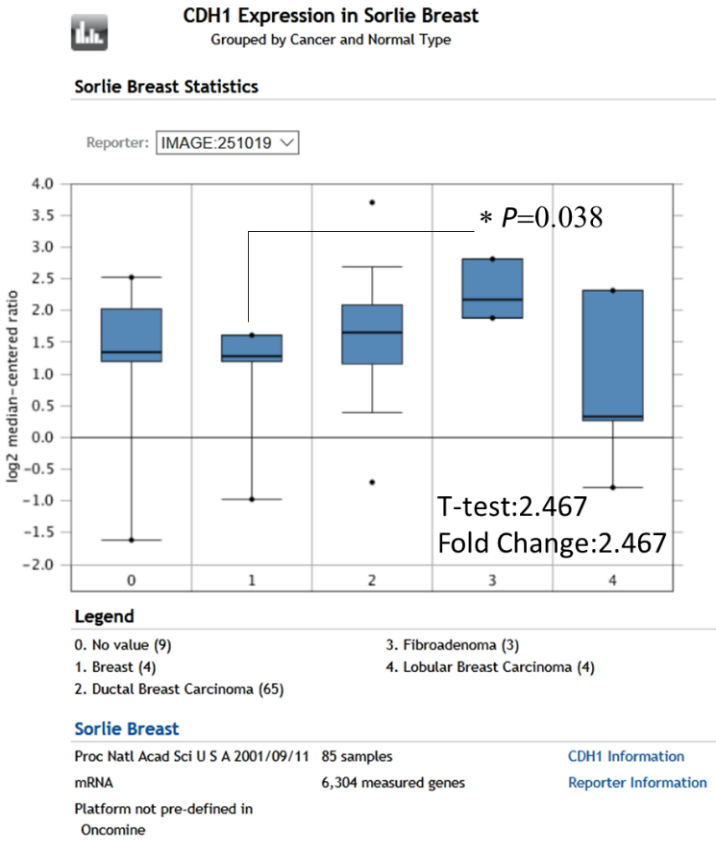

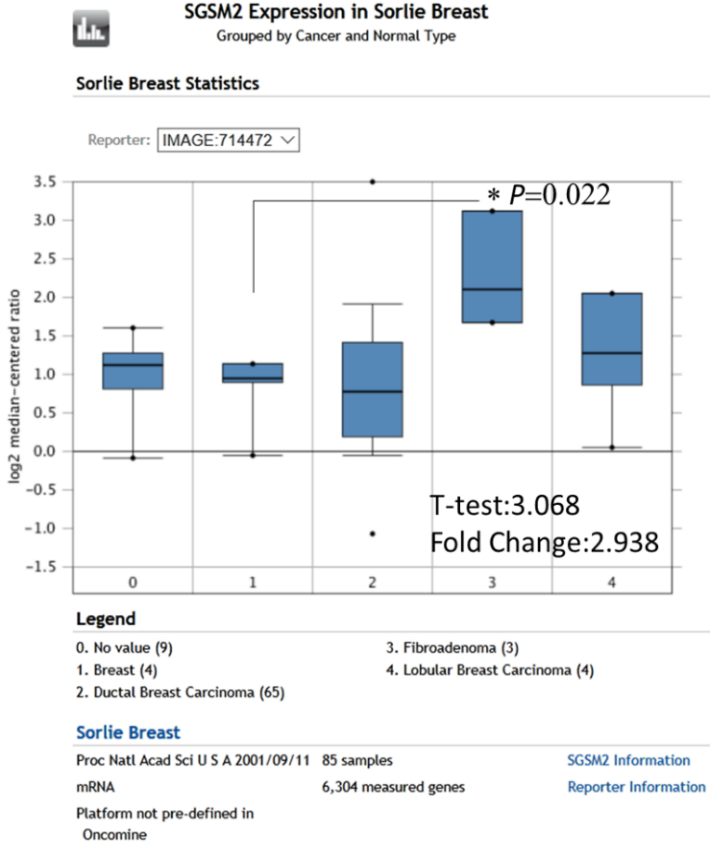


**A**

**B**

**C**

**D**

**Supplementary Figure S9. Identification of the correlation between *SGSM2* and *CDH1* in breast tumour tissues.** *SGSM2* expression was determined according to E-cadherin status in the (A) Turashvili Breast dataset and (B) Zhao Breast dataset using the Oncomine platform (<https://www.oncomine.org/resource/login.html>). (C) *SGSM2* and (D) *CDH1* expressions was the highest in the fibroadenoma category of the Sorlie Breast dataset. All P-values are two-tailed, and  indicates a P-value<0.05.


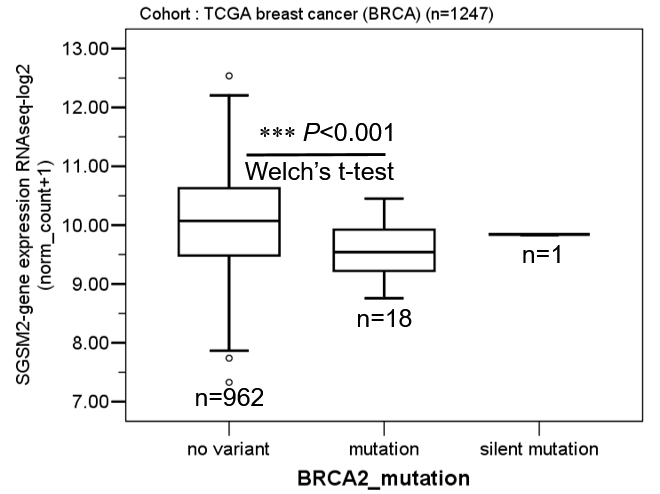

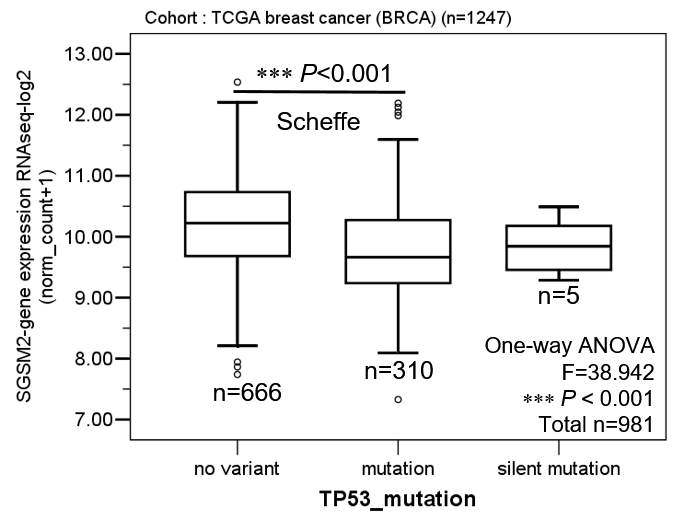


**A**

**B**

**C**


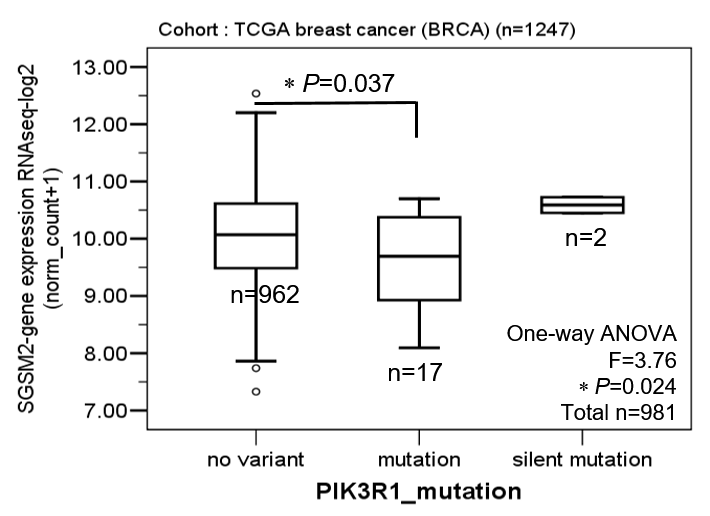


**Supplementary Figure S10. The correlation of *SGSM2* expression with TP53-, BRCA2-, and PIK3R1 mutation in breast cancer.** (A) Lower *SGSM2* expression was exhibited in TP53 mutation patients ( P<0.001), (B) in *BRCA2* mutation patients ( P<0.001), and (C) in *PIK3R1* mutation patients ( P=0.037) than in wild-type patients in the dataset from the TCGA Breast Cancer (BRCA) cohort using the UCSC Xena browser ([http://xena.ucsc.edu](http://xena.ucsc.edu/)). “n” indicates the number of cases.


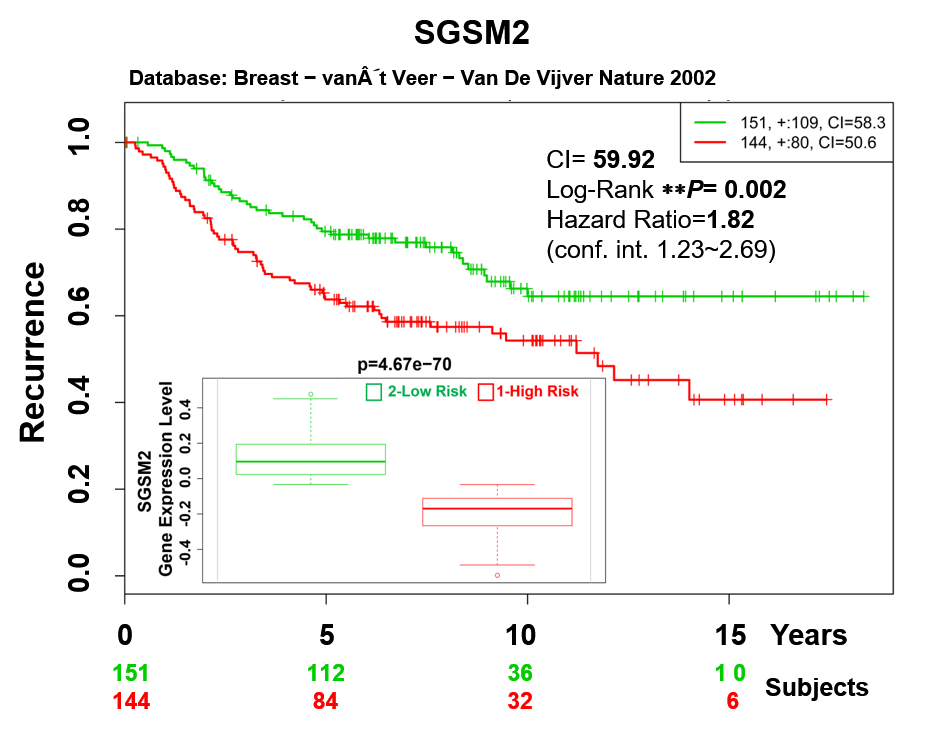

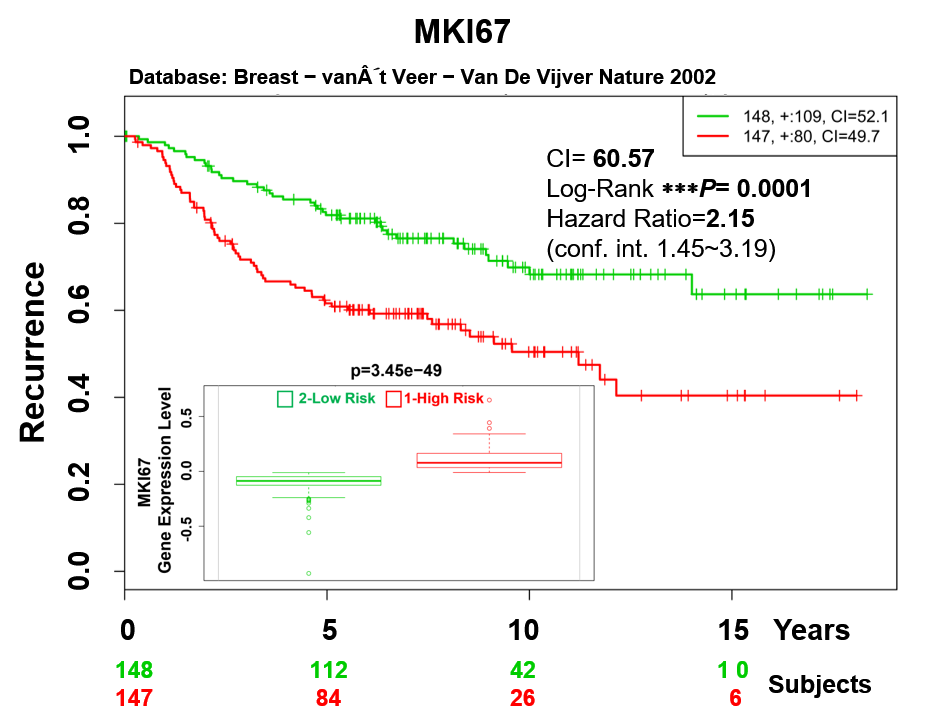

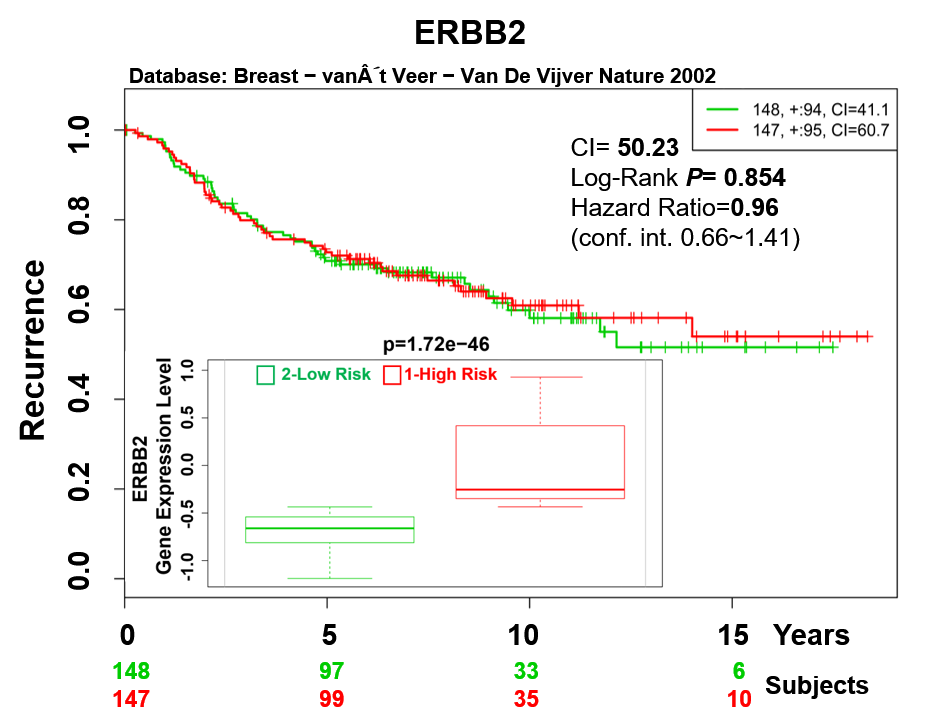

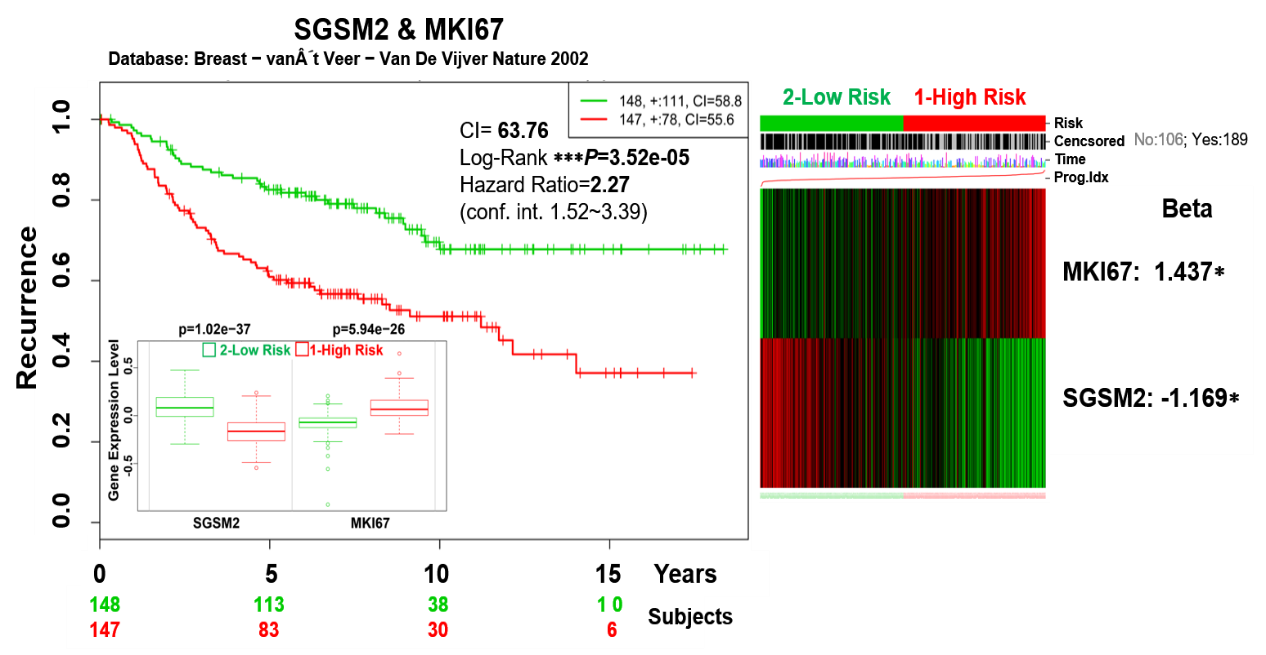

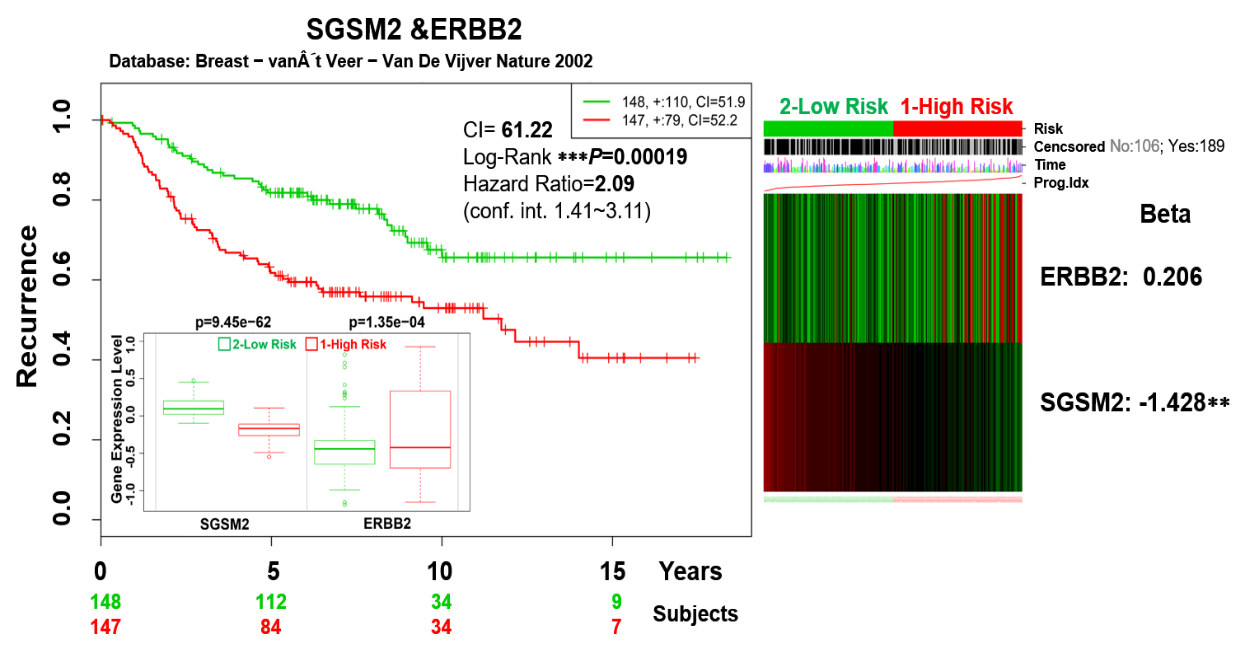


**A**

**B**

**E**

**C**

**D**

**Supplementary Figure S11. Recurrence outcome in breast cancer patients was predicted based on *SGSM2*, *ERBB2*, and *MKI67* expression levels.** Kaplan-Meier survival curves, Box plots, and heat map representation of the gene expression values generated using the SurvExpress biomarker validation tool ^8^ . These analyses used cohorts from datasets generated by vanÂ´t Veer − Van De Vijver Nature 2002. (A) BC patients with lower SGSM2 expression had higher risk of recurrence (HR, 1.82, conf. int. 1.23~2.69; P=0.002). (B) Box plots show higher ERBB2 expression in the high-risk group; however, there was no significant association between ERBB2 expression and recurrence outcome (HR, 0.96, conf. int. 0.66~1.41; P=0.854). (C) BC patients with higher MKI67 expression had significantly higher risk of recurrence (HR, 2.15, conf. int. 1.45~3.19; P=0.0001). (D) The two-gene (SGSM2 and ERBB2) combination was significantly correlated with recurrence in patients with BC (HR, 2.09, conf. int. 1.41~3.11; P=0.00019; Beta: ERBB2=0.026 and SGSM2=1.428). (E) The two-gene (SGSM2 and MKI67) combination was significantly correlated with recurrence in patients with BC (HR, 2.27, conf. int. 1.52~3.39; P=3.52e-05; Beta: MKI67=1.437 and SGSM2=1.169). “+”represents the censoring samples; “CI” is concordance index; “conf. int.” is the confidence interval; the horizontal axis represents time to event; green and red curves indicate low- and high-risk groups, respectively. The heat map shows the expression of each gene (rows) among samples (columns) in risk groups; low expression is represented in green, and high expression in red. Corresponding beta coefficients from the Cox fitting are shown Box plots compare the difference in gene expression between risk groups using a t-test.  indicates a P-value<0.05;  P-value<0.01, and  P-value<0.001.

**Supplementary references:**

1. Stark C, Breitkreutz BJ, Reguly T, Boucher L, Breitkreutz A, Tyers M. BioGRID: a general repository for interaction datasets. Nucleic Acids Res 2006; 34:D535-9.

2. Stelzl U, Worm U, Lalowski M, Haenig C, Brembeck FH, Goehler H, et al. A human protein-protein interaction network: a resource for annotating the proteome. Cell 2005; 122:957-68.

3. Huttlin EL, Bruckner RJ, Paulo JA, Cannon JR, Ting L, Baltier K, et al. Architecture of the human interactome defines protein communities and disease networks. Nature 2017; 545:505-9.

4. Ho HY, Rohatgi R, Lebensohn AM, Le M, Li J, Gygi SP, et al. Toca-1 mediates Cdc42-dependent actin nucleation by activating the N-WASP-WIP complex. Cell 2004; 118:203-16.

5. Guo Z, Neilson LJ, Zhong H, Murray PS, Zanivan S, Zaidel-Bar R. E-cadherin interactome complexity and robustness resolved by quantitative proteomics. Sci Signal 2014; 7:rs7.

6. Wan C, Borgeson B, Phanse S, Tu F, Drew K, Clark G, et al. Panorama of ancient metazoan macromolecular complexes. Nature 2015; 525:339-44.

7. Tsujita K, Suetsugu S, Sasaki N, Furutani M, Oikawa T, Takenawa T. Coordination between the actin cytoskeleton and membrane deformation by a novel membrane tubulation domain of PCH proteins is involved in endocytosis. J Cell Biol 2006; 172:269-79.

8. Aguirre-Gamboa R, Gomez-Rueda H, Martinez-Ledesma E, Martinez-Torteya A, Chacolla-Huaringa R, Rodriguez-Barrientos A, et al. SurvExpress: an online biomarker validation tool and database for cancer gene expression data using survival analysis. PLoS One 2013; 8:e74250.
